# Joint Modeling and Registration of Cell Populations in Cohorts of High-Dimensional Flow Cytometric Data

**DOI:** 10.1371/journal.pone.0100334

**Published:** 2014-07-01

**Authors:** Saumyadipta Pyne, Sharon X. Lee, Kui Wang, Jonathan Irish, Pablo Tamayo, Marc-Danie Nazaire, Tarn Duong, Shu-Kay Ng, David Hafler, Ronald Levy, Garry P. Nolan, Jill Mesirov, Geoffrey J. McLachlan

**Affiliations:** 1 CR Rao Advanced Institute of Mathematics, Statistics and Computer Science, Hyderabad, Andhra Pradesh, India; 2 Department of Mathematics, University of Queensland, St. Lucia, Queensland, Australia; 3 Division of Oncology, Stanford Medical School, Stanford, California, United States of America; 4 Baxter Laboratory for Stem Cell Biology, Department of Microbiology and Immunology, Stanford School of Medicine, Stanford, California, United States of America; 5 Department of Cancer Biology, Vanderbilt University, Nashville, Tennessee, United States of America; 6 Broad Institute of MIT and Harvard University, Cambridge, Massachusetts, United States of America; 7 Molecular Mechanisms of Intracellular Transport, Unit Mixte de Recherche 144 Centre National de la Recherche Scientifique/Institut Curie, Paris, France; 8 School of Medicine, Griffith University, Meadowbrook, Queensland, Australia; 9 Department of Neurology, Yale School of Medicine, New Haven, Connecticut, United States of America; Université Libre de Bruxelles, Belgium

## Abstract

In biomedical applications, an experimenter encounters different potential sources of variation in data such as individual samples, multiple experimental conditions, and multivariate responses of a panel of markers such as from a signaling network. In multiparametric cytometry, which is often used for analyzing patient samples, such issues are critical. While computational methods can identify cell populations in individual samples, without the ability to automatically match them across samples, it is difficult to compare and characterize the populations in typical experiments, such as those responding to various stimulations or distinctive of particular patients or time-points, especially when there are many samples. Joint Clustering and Matching (JCM) is a multi-level framework for simultaneous modeling and registration of populations across a cohort. JCM models every population with a robust multivariate probability distribution. Simultaneously, JCM fits a random-effects model to construct an overall batch template – used for registering populations across samples, and classifying new samples. By tackling systems-level variation, JCM supports practical biomedical applications involving large cohorts. Software for fitting the JCM models have been implemented in an R package EMMIX-JCM, available from http://www.maths.uq.edu.au/~gjm/mix_soft/EMMIX-JCM/.

## Introduction

Flow cytometry is widely used for single cell interrogation of surface and intracellular protein expression by measuring fluorescence intensity of fluorophore-conjugated reagents. Recent technical advances have taken the field towards single cell proteomics [1] and enabled highly multiparametric analysis [2] and computational cytomics [3]. Consequently, biomedical applications are presenting new challenges to cytometric analysis. Increasingly such studies involve cohorts with large numbers of patients, replicates, and may also use multiplexing of marker staining panels for probing large signaling networks [4]. Further, while typical flow experiments assayed for 4–8 features, the recent development of mass cytometry promises the ability to compare 50–100 features per cell [5], . Owing to multiple reasons such as variation among individuals in a cohort, simultaneous use of different stimulation conditions and panels in a given experiment, biological and technical replicates, the highly multivariate nature of the new platforms' measurements, etc., the resulting datasets are rich and complex. Currently there exists no single standard procedure for performing reproducible cohort-wide analysis while tackling systems-level heterogeneity and noise in multiple samples.

Recently, we developed a platform (FLAME) for automated analysis of high-dimensional flow data [7]. Each cell population (henceforth simply called population) in a sample is modeled by FLAME as a cluster of points with similar fluorescence intensities in the multi-dimensional space of markers. FLAME's heavy-tailed and asymmetric distributions are especially appropriate for flow data, since rare and interesting subpopulations tend to be represented by the tail-subpopulations that are connected to larger populations [8]. Notably, the field of computational cytomics has witnessed rapid growth in the past few years, as reviewed by Lugli et al. [3]

While modeling populations in flow data remains a difficult problem, a second and even more important challenge appears when there are many samples and conditions to compare – how to efficiently match or “register” the corresponding populations across a *batch* of samples. The difficulty of this problem arises from (a) the high-dimensionality of data, which prevents visual matching of populations, (b) large cohort or batch sizes, and (c) high inter-sample variation, all of which make the manual approach challenging. Yet it is essential to determine the batch-wise correspondence among populations with automation so that we can register them i.e., identify them uniquely, in high-dimension, which enables direct quantitative comparison of samples across conditions, phenotypes or time points. Addressed with algorithmic precision and rigor, automatic registration can facilitate clinical applications with diagnostic or prognostic implications. For instance, it can be useful for monitoring of specific cellular events such as lymphocytic infiltration in tumors, immuno-profiling of patients following treatment, etc. [9], [10]. By creating parametric models of the matched spatio-temporal profiles, we can use the estimated model parameters to accurately classify new samples as well as identify aberrant patterns (outliers).

A composite solution to these two complex problems – modeling each population within a sample, and registering them across samples – marks a significant improvement over FLAME and the other predominantly clustering approaches [3] such as flowClust [11] and SWIFT [12]. Currently, FLAME first models the populations separately within individual samples, and then tries to match these populations post hoc by running an external module (using Partitioning Around Medoids or PAM) on the model parameters. In our experience in running FLAME, this alignment procedure has several limitations. For instance, meta-clustering can be overly sensitive to the accuracy of the comparison results of PAM, which may be low if there is high inter-sample variation in a batch. Further, while PAM meta-clustering matches population-features only pairwise, the overall relationships among those features can be captured across all samples, i.e., in a manner more robustly against inter-sample variation, using batch-level modeling as in JCM. Finally, as the whole batch was not modeled simultaneously, no overall consensus template of the batch was formed by FLAME. In that sense, FLAME and other algorithms that analyze single samples cannot determine batch characteristics systematically.

### The JCM approach

We present a new multi-level framework called Joint Clustering and Matching (JCM) that operates on an entire batch of samples across two levels: (1) at a sample-specific “lower” level, JCM models every cell population as a cluster (i.e. a *component* of a finite mixture model of multivariate *t* or skew *t*-distributions); and simultaneously, (2) at a batch-specific “higher” level, JCM constructs a parametric *template*, which models overall characteristics of a batch. JCM achieves this by fitting a Random-Effects Model (REM) that allows every sample in a given batch to be modeled as an instance of an “original” template possibly transformed with a flexible amount of variation. In Appendices S1 and S2, we describe our Expectation-Maximization (EM) algorithm for efficient fitting of the two-level JCM model, as described in (1) and (2) above. Its multi-level design gives JCM the ability to establish a direct parametric correspondence between each cell population in the batch template and its counterpart within an individual sample. Unlike FLAME, this allows JCM to explicitly tackle inter-sample variation, a common concern for flow data, and thus support both biological and clinical applications. JCM's template based mixture-model approach was described originally in our unpublished working paper [13].

In recent years, researchers have also started multiplexing many staining panels to overcome limits on the numbers of markers that can be accurately measured together using commercial cytometers [4]. While the resulting data are more enriched, it can also produce a large number of distinct features from every panel of markers. Currently there exists no technique for systematic integration of such features across panels into meta-features for the common underlying sample. As part of JCM analysis, we introduce a new technique to combine both univariate and multivariate JCM features across multiplexed panels to construct enriched meta-features (or *feature-sets*), and use these to improve sample classification.

Using simulation as well as several real-world benchmark datasets, we found that key performance attributes such as classification accuracy and running time of JCM are quite favorable compared to other methods. To illustrate the different capabilities of JCM, we applied it to two sets of experiments involving multiple markers, time points (or stimulations), staining panels, and sample classes. In addition, the accuracy of JCM is compared with FLAME and HDPGMM on a set of manually analyzed benchmark DLBCL datasets from the flowCAP contest [14]. Here, HDPGMM denotes the hierarchical Dirichlet process Gaussian mixture model-based procedure proposed recently [15]. The procedure provides a strategy for the alignment of cells across multiple samples by assuming the cell populations to have identical location and shape across the samples, but their weights (or proportions) may vary from sample to sample. Similar to JCM, the HDPGMM is an alternative procedure that produces a template or consensus model to represent the overall distribution of the batch of samples. However, the assumption of identical mean and covariance in the component normal distributions for all samples may be too restrictive in some cases. We also compared JCM with two other popular methods for the automated analysis of flow cytometric data, namely flowClust and SWIFT. As a model-based algorithm, flowClust also uses mixture models for density estimation and clustering, but adopts a data transformation approach to handle asymmetric clusters as an alternative to merging Gaussian mixture components (HDPGMM) or adopting a skew component distribution (FLAME and JCM). One advantage of the former approach is a potentially faster run time due to a simpler model fitting procedure. SWIFT is closely related to HDPGMM in that they are both based on merged Gaussian mixture models, but the former is also designed for scalability to larger datasets by employing weighted down-sampling to speed up model fitting. However, as these two methods do not have any explicit facility for matching the output from a series of samples, we applied them to each sample considered separately and to the single sample consisting of all 16 samples pooled into one.

Concerning the setting of several parameters here in our analyses, we note that it is in fact the biologist who decides the number (and types) of markers necessary for characterizing the populations of interest before the data are generated. Given the generated data, the JCM algorithm allows automated estimation of all the parameters of the fitted JCM model in an unsupervised manner, that is, with no explicit need of manual setting of the model parameters. In the two sets of experiments performed to asses JCM, we applied JCM to obtain multi-parametric characterization of different T cell subpopulations upon T cell receptor (TCR) stimulation in a time course phosphorylation experiment. This illustrates how a complex multi-class and multi-sample experiment can be systematically analyzed in a fully automated and reproducible manner to generate precise and objective profiles for every class. Importantly, it is based on a comprehensive list of rigorously estimated model parameters for each population, which is output by JCM. As illustrated by our next application, such unsupervised, thorough approach can also reveal new or subtle expression phenotypes in specific subpopulations, which might otherwise go undetected in manual gating. In the second experiment, we applied JCM to understand differential patterns of altered B cell receptor (BCR) signaling in human follicular lymphoma (FL) tumor samples. By combining JCM features from multiplexed panels of 16 phospho-markers, we identified a novel spatio-temporal signature of BCR signaling in a specific subpopulation of the lymphoma B cells that improved the separation between two classes of patients previously reported by Irish et al. [9] to have markedly different survival. We also devised visual means for overlaying expression templates to capture the variation in data both within and across a batch. This highlights the capability of JCM to distinguish complex biological contexts via quantitative class-specific characteristics, which may be very useful in new studies involving large cytometric cohorts.

## Results

### Spatio-temporal characterization of TCR activation

We analyzed phosphorylation patterns downstream of T cell receptor (TCR) activation in naïve and memory T cells across six classes of samples corresponding to six time points: 0, 1, 3, 5, 15, and 30 min originally measured by Maier et al. [16]. In that study, human expertise played a key role in manually and visually identifying each population in every sample at every time-point, and then carefully comparing them based on selected features of chosen populations. In the process, many manual decisions were taken and highly supervised time-consuming operations were performed repeatedly such as the applied sequence of gates, the selection of useful parameters for comparing the subsets across classes, etc. Traditionally, therefore, the results of manual gating even on similar experiments can vary with such decisions, which in turn depend on the experience of the human expert.

JCM, in contrast, produced the full sequence of spatio-temporal expression phenotypes of phosphorylation in five distinct subsets of T cells, which are matched across all samples. These five populations were characterized in a fully unsupervised manner in 4-dimensional marker-space, as well as in terms of the 5*^th^* dimension of time. The model yielded a comprehensive list of matched high-dimensional parameters, not just a few pre-determined visual (i.e. 2-D) features. This list could be readily used for exploratory statistical analyses (e.g. feature selection, discriminant analysis) to accurately identify the changes in every population over time. Since the cohort was modeled as a batch by JCM, we can also compare the overall batch-templates computed for every time-point, both statistically and visually, to capture the longitudinal phenotypic trend starting from the activation of TCR up to its de-activation. Thus the JCM framework is objective, fast, quantitative and reproducible.

**Figure 1:**
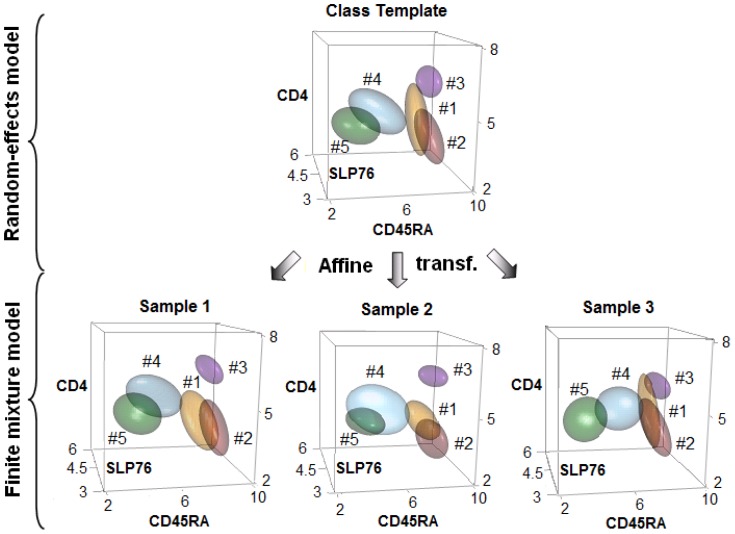
JCM model and application. The multi-level model is illustrated using the samples (bottom) and the template (top) for the samples of the 3 min class, along 3 out of 4 dimensions in the TCR activation data. Actual values of the JCM parameters were used to construct the 50^th^ percentile multivariate *t* density contour (ellipsoid) depicting every population. The overall class template is computed by fitting a random effects model on all the samples, which in turn are fitted with sample-specific finite mixture models of multivariate *t*'s. Under the JCM framwork, each sample can be described as an affine transformation of the template, where each population in a sample corresponds to its counterpart in the class template, as shown by the matched colors and labels (# 1–5).

The sequence starts at 0 min, prior to stimulation with an anti-CD3 antibody (baseline measurement), reached peak levels of phosphorylation at 3–5 min, then subsided by 30 min. JCM's multi-level modeling of the time course data is illustrated in Figure 1 (for the time point of 3 min), where each sample is modelled as an instance of the class template through an affine transformation, thus inherently aligning the cell populations across different samples. In particular, the transformation is governed by a REM (see *Methods*). This allows JCM to flexibly accommodate subtle variations between the samples and facilitates interpretability of the results. The profile of each of the five populations (denoted #1–5 in Figure S2) were distinguished apart, matched across samples, summarized with templates and compared across six time-points. The overall changes summarized as high-dimensional templates for each of the successive classes can be observed in Figure S2. Looking at the changes in the proportions of the five clusters (denoted by *π*_1_ to *π*_5_; see *Methods*) over the six time points, we can see from Figure S2 that the estimate of *π*_3_ is relatively constant, while the estimates of *π*_1_ and *π*_5_ are on the increase and the estimate of *π*_4_ is on the decrease. The overall spatio-temporal differences both within and across classes may be observed with JCM's overlay plots (Figure S3). Specifically, the alterations in the naïve and memory T cell populations are outlined in Figure S4, where a rise in the intensities of marker ZAP70 can be observed soon after stimulation and then a gradual decline over time. For details on the experiments, see Text S1.

Two markers in the staining panel, CD4 and CD45RA, were used for characterizing the different populations, while two other markers, SLP76 (p-Y128) and ZAP70 (p-Y292), were used to measure the intensity of phosphorylation in these subsets. As described in Maier et al. [16], we used the signatures 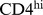 with 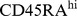 and 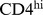 with 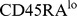 to represent the primarily naïve and memory T cell subsets, respectively. Upon fitting mixtures of *t*-distributions to each of the 6 classes, an overall pattern for five matched populations emerged (indexed 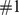 through 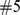 in Figure S2A–E). As expected, a rapid rise in the intensities of phosphorylation markers SLP76 and ZAP70, especially the latter, was observed soon after stimulation for all populations with the possible exception of 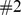. While both naïve (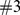) and memory T cell subsets (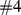) showed similar peak levels of phosphorylation initially (Figure S2C–D), the former exhibited a faster decline with time (Figure S2D–E), consistent with prior results [1]. In fact, both 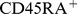 populations (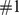 and 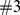) exhibited similar expression throughout. Upon p-CD3 (p-Y142) normalization, higher phosphorylation in memory T cells compared to naïve T cells between 5 and 15 min – as observed manually [16] – was recapitulated with help of JCM.

### BCR signaling feature-sets distinguish FL subclasses

In a recent study based on human expert analysis, Irish et al. [9] stratified follicular lymphoma (FL) patients into two classes with markedly different overall survival depending on the presence or absence of a Lymphoma Negative Prognostic (LNP) subset of B cells in tumor. The LNP cells showed altered BCR signaling, and were identified by the expressions of a multiplexed panel of selected phospho-markers. The multiplexing of markers, used for assaying each sample with a large set of markers (too large to be contained in a single panel) that is distributed across multiple panels, is described in detail in Irish et al. (Fig. 1A and Supplementary Information in [9]). The signaling based stratification of patients into 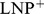 and 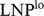 classes is therefore of clinical significance. We used JCM for (a) automation — to systematically combine features from multi-panel data from FL patients, and (b) discrimination — to identify features that could separate the pre-defined FL patient classes as best as possible.

In the BCR signalling dataset, through automated analysis of multiplexed data, JCM had identified a nuanced signature for signaling alterations in high-dimensional marker-space that further improved the stratification between the two FL patient classes, as described in Irish et al. [9]. The difference between the two classes was determined by comparing the class meansusing the *t* test. We analyzed 28 pre-processed patient samples for two time points, 0 min and 4 min (i.e. pre- and post-BCR stimulation, respectively). Further details of the samples and preprocessing are provided in Text S1.2 and S2. At every time-point, and for all patients, the data for each sample was available for eight multiplexed panels, each with results for four markers, including two B cell markers CD20 and BCL2 that were common to every panel. Signaling responses were measured in terms of phosphorylation of 16 phospho-proteins from the BCR signaling network. By multiplexing panels, the signaling for all these network components could be measured in every sample. Each sample's phenotype (or class label), 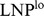 (18 samples) or 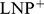 (10 Samples), was assigned by human expert analysis (Supplemental Methods of Irish et al. [9]).

For both unstimulated (0 min) and stimulated (4 min) conditions, each class of patient samples was modeled with an overall template produced by the JCM procedure using two-component multivariate skew *t*-mixture models. The templates revealed the class-specific features of two lymphoma B cell populations. For convenience, let us call these two populations “mound” and “base” corresponding to higher and lower levels of stimulation respectively. These are components of the JCM mixture model that primarily represent populations in which BCR signaling is intact (i.e. non-LNP cells) as opposed to altered (LNP cells). The change between the corresponding features pre- and post-stimulation provided a kind of baseline correction to the resting level of signaling for each sample. This approach corresponds to asking whether the response of lymphoma B cells to BCR engagement was heterogeneous, but using the entire set of continuous features for exploring tumor heterogeneity rather than only median phosphorylation, the primary discretized feature in the Irish et al. study [9].”

**Figure 2:**
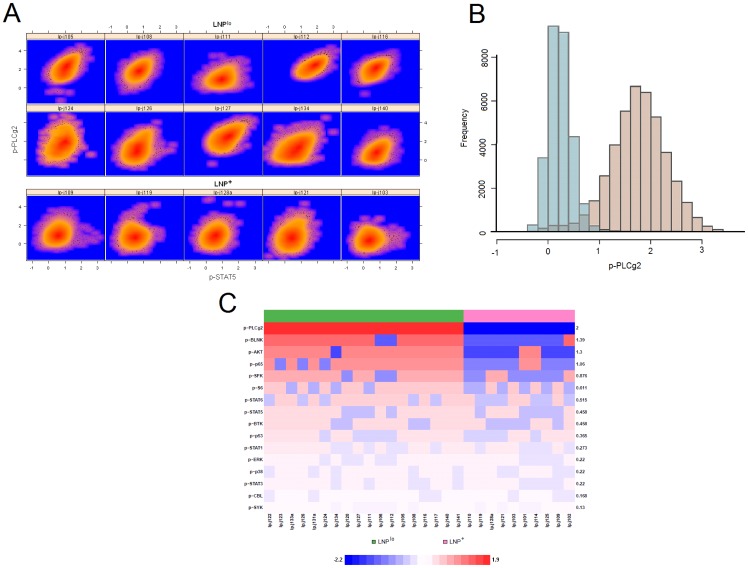
Distinct spatial characteristics of phospho-marker expression in samples from two classes of patients with different outcomes. (A) Heatplots provide insight into the distribution of phospho-proteomic expression of p-PLCg2 and p-STAT5 (panel 4) for 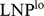 (top 2 rows) and 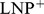 (bottom row) samples. The mound (high CD20 and BCL-2) populations are shown here. In contrast to the more symmetrically distributed, well-rounded 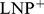 mounds, the skewness in the 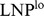 mounds is clearly visible. (B) The stimulated mound (light brown histogram) of a 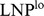 sample is shown in contrast with the corresponding population prior to stimulation (greyish blue histogram). (C) The ability of the mound skew parameters (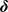) for 16 phospho-markers to distinguish samples across the 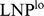 and 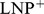 classes (green and pink labels respectively) is shown with a heatmap based on the corresponding posterior log-odds scores. The higher the score, the darker the corresponding entry in red/blue. Each marker name and its average posterior log-odds score over all samples are marked on the sides of the heatmap.

We introduced a new strategy for a combined analysis of multiplexed markers probing different parts of the BCR signaling network. The JCM features of 16 phospho-markers distributed across all 8 panels were pooled to form an enhanced meta-feature, or a feature-set, that is analogous to the concept of a gene-set (GSEA [17]). Thus we applied Gene Set Enrichment Analysis (GSEA [17]) to every feature-set to test their abilities to distinguish between 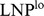 and 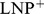 samples. Notably, Irish et al. [9] had previously discovered that the size of the LNP population could be used to distinguish FL patients into two classes with different outcomes. However, these results were based on manual demarcation of the LNP subset, and therefore based on low-dimensional gating of data. Interestingly, in our feature-set enrichment analysis, the single most significantly enriched feature-set (at *P*-value level 0.05 by Kolmogorov-Smirnov test of GSEA [17]), i.e. the most distinctive meta-feature across these two patient classes, was skewness (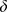) of the mound at 5 min. (*P*-value 0.0144, *q*-value 0.058; Figure S5). Across 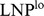 and 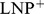 classes, this spatial signature (i.e. stimulated mound skew) is distinctive both visually (Figure 2A and 2B) and statistically (the average of posterior log-odds ratios in Figure 2C, computed using Bayesian methods described in [18], particularly for markers such as p-PLCg2, p-BLNK, and p-SFK (Figure S6). In particular, we draw attention to Figure 2A, outlining the asymmetric expression of the mound in LNP^lo^ samples, which contrasts with their more spherical counterparts (i.e. lower skew) in the 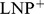 samples. The distinction is in fact statistically significant even after controlling for the corresponding base (LNP) 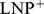 population sizes (e.g. for p-SFK the GLM based p-value after controlling is 0.0079).

The skewness, given by the parameter vector 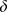, of the stimulated mound in 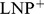 samples is expressed in the form of a heavy left tail (Figure 2B). This suggests the likely presence of a subpopulation of primarily non-LNP cells with partially altered signaling at a given time-point. Whether it is of real prognostic value needs to be tested in future studies. Our main point is that JCM's automatic feature detection can reveal new spatio-temporal states and their characteristics. State transitions can be numerically measured and monitored even if they are subtle across classes. For instance, if the alteration in BCR signaling happens in a way that is gradual and not sharp, then it can be difficult to demarcate or determine the size of the LNP component accurately, and yet the skew feature can be used for nuanced understanding of the change in the same population thus providing mechanistic insights into the biology of the system in action.

### Cell population identification and alignment across DLBCL batch samples

We compared JCM with two other flow analysis methods that compute cluster correspondence, namely, FLAME and the HDPGMM procedure. As with JCM, FLAME is based on mixtures of skew *t*-distributions, while HDPGMM uses mixtures of normal distributions. Note that although the HDPGMM model adopts the multivariate normal distribution as component distributions, it has some flexibility in handling clusters that are not distributed normally in that it can use more than one normal distributions to model the distribution of observations in a cluster. Based on a real-world benchmark dataset from the flowCAP1 contest [14], we compare the performance of JCM with several other competing procedures in cell population identification and alignment across a batch of samples. In the original dataset, 30 samples were collected from patients diagnosed with diffuse large B-cell lymphoma (DLBCL). For this illustration, we use the subset of 16 samples which were manually analyzed and were determined to have the same number of clusters. With JCM, we first created a template across the batch of 16 samples. Then the cluster membership labels given by JCM for each sample are compared with the results given by manual gating. The results are given in Table 1, along with the corresponding results for FLAME and HDPGMM procedures.

**Table 1:** Classification error rates of three methods on DLBCL data.

Sample	JCM	HDPGMM	FLAME
Sa001	0.3045	0.2046	0.5143
Sa002	0.0339	0.1044	0.4300
Sa003	0.0694	0.0946	0.5931
Sa004	0.0659	0.0946	0.5459
Sa005	0.0089	0.1230	0.4440
Sa006	0.2947	0.0611	0.5987
Sa007	0.0208	0.0510	0.2584
Sa008	0.0683	0.0719	0.3719
Sa009	0.0249	0.1343	0.2417
Sa010	0.0121	0.3828	0.5413
Sa011	0.0236	0.4082	0.4792
Sa012	0.0096	0.1148	0.2456
Sa013	0.0326	0.3247	0.5947
Sa014	0.0062	0.2959	0.6000
Sa015	0.1283	0.4110	0.3927
Sa016	0.0361	0.4437	0.5372
AMCR	0.0711	0.2038	0.4618

Samples from 16 patients diagnosed with Diffuse Large B-cell Lymphoma (DLBCL) were clustered using JCM, HDPGMM, and FLAME. For both JCM and HDPGMM, a class template is computed for the entire batch of samples, while FLAME performs post hoc alignment of the results given by FLAME-I, where FLAME-I denotes the procedure with FLAME applied to each individual sample considered separately. The final row shows the average misclassification rate (AMCR) for each method. Clearly, JCM shows overall superior performance.

In 14 of the 16 samples, JCM achieved the lowest misclassification rate (MCR) among the methods. This MCR is calculated for each permutation of the cluster labels of the clustering result under consideration against the class labels given by manual expert gating and the rate reported is the minimum value over all such permutations. For reference, we have included in Table S1 the corresponding results using the *F*-measure as reported in [14], which is given by the harmonic mean of precision and recall. Our discussions here will focus on the MCR, which is the standard rate used in statistics to assess the performance of classifiers and also clustering procedures in studies where the true labels are known. However, we note that the relative ranking of the methods remains similar using Table S1.

JCM's average MCR of 0.0711 is well below the average rates of 0.2038 and 0.4618 for HDPGMM and FLAME, respectively. It can observed from Table 1 that JCM had a lower MCR than FLAME for all 16 samples, and also in 14 of the 16 samples when compared to HDPGMM. For the two samples, Sa001 and Sa006, on which it does not have the lowest MCR, its performance is well below what it is for the other 14 samples. Given the presence of these two samples with atypically high MCRs, we computed the median MCR of JCM for these 16 samples. It was only 0.0333, being just under half the average MCR. As mentioned in the introduction, FLAME adopts a single-sample based approach to the analysis of multiple samples, and so it does have its limitations in registering the individual results across the samples. This is clearly evident in Table 1, where the MCR for FLAME is quite high relative to JCM and HDPGMM which analyse the samples simultaneously.

We have also listed in Table 2 the MCR for each of the 16 samples clustered according to FLAME-I and FLAME-P, where FLAME-I denotes the procedure with FLAME applied to each individual sample considered separately and FLAME-P denotes FLAME based on the single sample formed by pooling the 16 samples together. If there were little inter-sample variation, then one would expect FLAME-P to be similar or even superior in performance to JCM. But it can be seen from Table 2 that JCM has a lower MCR than FLAME-P except for only three samples that include the aforementioned two samples (Sa001 and Sa006) on which JCM performs poorly. The MCR for JCM is also lower than that for FLAME-I except for only three samples (apart from Sa001 and Sa006). For these three samples, the differences between the MCR for JCM and FLAME-I is zero up to the fourth decimal place.

**Table 2:** Classification error rates of various methods on DLBCL data.

Sample	JCM	FLAME-I	flowClust-I	SWIFT-I	FLAME-P	flowClust-P	SWIFT-P
Sa001	0.3045	0.3039	0.3070	0.5368	0.1666	0.2187	0.3039
Sa002	0.0339	0.3394	0.0388	0.1526	0.2146	0.4096	0.3060
Sa003	0.0694	0.0753	0.0588	0.4500	0.1790	0.3194	0.2204
Sa004	0.0659	0.0687	0.0682	0.5506	0.1227	0.1661	0.3038
Sa005	0.0089	0.1631	0.1868	0.4521	0.1415	0.0752	0.1220
Sa006	0.2947	0.2670	0.1150	0.3612	0.0809	0.3773	0.1869
Sa007	0.0208	0.0211	0.0217	0.2580	0.0943	0.0569	0.0438
Sa008	0.0683	0.0678	0.0997	0.1911	0.0852	0.1045	0.3560
Sa009	0.0249	0.3191	0.0891	0.2508	0.0487	0.0302	0.0186
Sa010	0.0121	0.0575	0.0111	0.5353	0.0628	0.0471	0.0757
Sa011	0.0236	0.0248	0.0248	0.1627	0.0240	0.1660	0.1004
Sa012	0.0096	0.3919	0.4613	0.2170	0.0421	0.0299	0.0188
Sa013	0.0326	0.0324	0.0355	0.5936	0.0796	0.0500	0.0581
Sa014	0.0062	0.0065	0.0083	0.5612	0.0857	0.0159	0.0373
Sa015	0.1283	0.1274	0.1317	0.5896	0.1093	0.1077	0.0947
Sa016	0.0361	0.0554	0.1832	0.4502	0.0524	0.0535	0.0803
AMCR	0.0711	0.1451	0.1151	0.3946	0.1128	0.1393	0.1454

Misclassification rate (MCR) for JCM, FLAME, flowClust and SWIFT on the 16 samples from the DLBCL dataset (see also Table 1). The latter three methods were applied to each individual sample separately (denoted with suffix -I), and also based on a pooling approach (denoted with suffix -P). The final row shows the average misclassification rate (AMCR) for each method.

For comparative purposes, we have also included in Table 2 the corresponding MCR for these 16 samples clustered according to two other methods in flow cytometry, SWIFT and flowClust. As these two methods do not have any explicit facility for matching the output from a series of samples, we reported the MCR for SWIFT-I and flowClust-I corresponding to SWIFT and flowClust applied individually to each sample and for SWIFT-P and flowClust-P corresponding to SWIFT and flowClust based on the pooled sample. It can be seen from Table 2 that for the 16 samples FLAME-I and flowClust-I have similar performances for most of them as do FLAME-P and flowClust-P. For example, FLAME-I has a lower MCR than flowClust-I in 9 of the 16 samples, with there being one tie between FLAME-I and flowClust-I. The flowClust method fits mixtures of *t*-distributions after first applying a Box-Cox transformation. We note that if the transformation is sample-specific, then this approach of first transforming each sample considered separately makes it difficult to compare the differences between the fitted distributions for a series of samples corresponding, for example, to different patients or to the one patient monitored over a series of time points. Concerning the SWIFT procedure, it can be seen from Table 2 that SWIFT-I has a higher MCR than FLAME-I and flowClust-I for most of the samples. However, the average MCR (AMCR) for SWIFT-P is much closer to that for FLAME-P and flowClust-P. Indeed, SWIFT-P has a lower MCR than JCM for three of the samples, including the two samples for which FLAME-I and FLAME-P was performing better than JCM. On comparing flowClust and SWIFT with JCM, it can be observed from Table 2 that JCM had a lower MCR for all samples than SWIFT-I, and in 13 and 14 of the 16 samples compared to flowClust-P and flowClust-I, respectively. Overall, JCM is clearly favoured by both MCR and the *F*-measure in this dataset, as evidenced by it being ranked first or second in 13 of the 16 samples among the five methods based on both MCR and the *F*-measure.

## Discussion

High-dimensional computational analysis of flow data is receiving increasing attention with the rapid rise in the number of markers that can be used to probe each cell in parallel [3], [6]. By mirroring the perception of a flow sample as a mixture of cell populations, finite mixture of Gaussians has long been an attractive modeling mechanism [19]. Recently, robust mixture models with multivariate *t* and skew *t* distributions were introduced for analyzing flow data with non-Gaussian features such as outliers, heavy-tailed densities, and asymmetric shapes [7], [20]–[22]. In addition to modeling the cell populations, Pyne et al. [7] also highlighted the importance of registering them across samples. Recent studies have noted that for re-structuring of cell populations, the optimal algorithmic strategy is to do so in conjunction with population modeling [20], [22].

The key contribution of JCM is its joint approach to address two challenges with a single composite model. It is a two-level framework for simultaneous mixture modeling and registration of populations in an entire batch of flow samples. That allows JCM to meet a key need of cytomics – reproducible analysis of data from many samples and conditions simultaneously. Notably, in the field of pattern recognition, alignment of images and curves in lower-dimensional space have emerged as active areas of research in recent years [23]–[25]. Thus, JCM provides an important extension from Gaussian mixture regression models [24] to multivariate *t*- and skew *t*-models, which can be fitted via the EM algorithm. This algorithm is an effective generic technique for parameter estimation [25], and we have extended it for the JCM-specific application of EM (Appendices S1 and S2). Thus the JCM framework is objective, fast, quantitative and reproducible.

As demonstrated in the previous section, automated population registration of JCM marks a significant technical improvement over FLAME. Unlike the post-hoc meta-clustering approach of FLAME, matching of populations by JCM is intrinsic to its modeling strategy. It is achieved by fitting a random-effects model (REM), a meta-analytic approach for estimating the mean of a distribution of effects [26]. Rare past usage of REM in cytomics was limited to measuring variability of very specific features, e.g., CD4 expression [14]. JCM is perhaps the first framework that incorporates REM for comprehensive batch characterization in flow data analysis (Figure 1). In particular, our REM uses affine transformation parameters to explicitly learn relationships among every population in a batch even in the presence of flexible amounts of cross-sample variation. In theory, were JCM to be reduced to its lower level, i.e., to perform clustering only and restricted to just a single sample input, then it would be equivalent to FLAME clustering. FLAME was ranked by rigorous benchmarking and expert analysis to be among the top performing unsupervised algorithms at a recent international contest on flow analysis FlowCAP1 organized in NIH [14]. This signifies that JCM has much greater potential with its more flexible approach compared to FLAME.

A technical advantage of JCM's REM-based registration is that it accounts for the populations' scaling and shifting transformations without explicitly “correcting” them. Some programs may shift populations in order to apply a common gate or filter on an entire cohort, without considering inter-sample variation. However, for precise modeling of the populations, we want to identify those spatio-temporally distinctive high-dimensional features, which may actually be characteristic of each individual sample's phenotype. Whereas we do not want to homogenize population features by aligning them, at the same time, we do want to register the populations – as they appear in high-dimensional space – with precision and rigor. This makes registration more challenging than just matching (as in FLAME meta-clustering [7]) or alignment (as in channel normalization [27]). In fact, we compared the performances of JCM and FLAME meta-clustering on benchmark data and, as shown in Table 1 JCM with its use of a template keeps classification error rates low in the face of increasing inter-sample variation in batches derived from real cytometric cohorts.

Perhaps the most attractive feature of REM is an overall consensus template that emerges from connecting both levels of the JCM model (Figure 1). Thereby JCM establishes a direct parametric correspondence between each population in the batch's template and its counterpart within every sample. Further, the template allows JCM to capture across-sample inter-relationships that may exist among populations and are useful for accurate registration. For instance, if a certain population A usually appeared in between two populations B and C, then it is useful to learn about such relative positioning of A even if its actual location varied from sample to sample. It makes JCM more robust to common transformations (such as shifting or scaling of populations – to which these relationships are generally invariant) compared to FLAME meta-clustering, which can handle only limited variation in actual locations. Thus the JCM template provides a “ground truth” while the REM transformation parameters quantify each individual instance's deviation from that reference structure. From classification standpoint, given that the JCM templates are defined by parametric distributions, they allow direct statistical comparison of batches which could represent, say, different subclasses of patients or successive longitudinal observations. We also present overlay plots for visual comparison of overall batch-structures along every dimension both within and across different classes in Figures S3 and S4. Moreover, any new patient sample can be easily classified with the group that has the most similar template (as determined by, say, Kullback-Leibler distance). Finally, a JCM template provides the user with a visually convenient yet parametrically precise “snapshot” summarizing a cohort's overall population structure. Studies of large cohorts, such as for finding associations between genotypes and immuno-phenotypes in human populations, can be performed systematically with our two-level approach. Thus large population-wide immune cytome databases can be created.

Parametric characterization of cohorts in terms of their high-dimensional spatio-temporal features can reveal complex and dynamic biological contexts and present them for further investigation. Dissecting and monitoring the parameters of individual cellular species as they evolve over time — such as our time course profiling of TCR stimulation (Figure S2) — could be useful in many biomedical applications. The JCM models supporting asymmetric and heavy-tailed distributions of events are uniquely suited for detecting features that appear dynamically as hard-to-separate transitional features, such as asymmetric or tail subpopulations [8], that are otherwise difficult to distinguish via automation. Further, by pooling features across staining panels that are multiplexed, JCM can detect complex biological contexts involving multiple markers from a signaling pathway or network [9], which is a new application in computational cytomics.

JCM can serve as a practical framework that is suitable for clinical applications. Here, its main objective is to learn the specific target populations' parameters for large numbers of samples precisely and quickly. Yet, in clinical applications, the modeling must also be robust enough to allow a reliable parameter-driven classification of patient samples. This is of particular concern for flow data which may contain high inter-sample variation due to the presence of complex, biologically interesting subpopulations, along with noise, within the target pool of primary cells. In the BCR signalling dataset, through automated analysis of multiplexed data, JCM had identified a nuanced signature for signaling alterations in high-dimensional marker-space that further improved the stratification between the two FL patient classes, as described in Irish et al. [9]. Explicit detection of variation by REM is useful for batch characterization, QA/QC, as well as downstream analysis.

Moreover, JCM produces an array of insightful plots. For instance, the overlay plot can reveal within-class variation along any dimension (Figure S3), while the intensity heatplots take advantage of REM to allow monitoring of spatio-temporal changes in individual populations that are matched across the cohort (Figure 2). Another attractive practical feature of JCM is its representation of output in the form of a generic feature-by-sample matrix, which can be analyzed with common bioinformatic pipelines. Thus, here we used the well-known GSEA algorithm [17] to create a new technique for combining JCM features into enriched meta-features across multiplexed staining panels. The simple new technique may become highly effective as more multiplexed staining data begin to appear [4].

By accounting for sample-specific variation, in essence REM also performs cohort-wide meta-analysis. Indeed, JCM framework can be further generalized to include an even higher level of parameterization for representing class-specific information such as time points or patient subtype (including clinico-pathological variables, genotypes, etc.). This makes JCM well suited for integrative cytomics, such as for large population immunome studies. In fact, our simulations show that besides being efficient in batch mode analysis, JCM is also robust against both class-size and the amount of inter-sample variation it can handle (Figure S7). In particular, we conducted an extensive set of simulation studies to determine the performance of JCM under different settings, including Simulations A to D reported in Figure S7 which focus on the performance of JCM with different number of sample sizes, markers, populations, and samples (in a cohort), respectively. Simulation shows that the run time performance is linearly proportional to the number of samples, the number of observations per sample, and the number of clusters. For instance, the running time for JCM modeling of a sample in our phosphorylation data averaged 33.7 sec per sample on a standard desktop PC (again using only a single-threaded implementation of JCM). This contrasts sharply with the hours of manual analysis performed over weeks by multiple researchers in the original study. With increasing multi-parameterization and multiplexing of cytometric data, JCM can facilitate automated, quantitative, scalable and objective investigation of complex hypotheses about different conditions and cohorts of biomedical interest.

## Methods

Following is the description of the JCM workflow and details of the models and methods, also continued in Text S2.

### Overview of JCM

JCM is run in the following sequence of steps (flowchart in Figure S1) –

1. Obtain the expression matrices from an input batch of preprocessed samples.
2. Fit a two-level model (as illustrated in Figure 1) to these data such that —
**(2a)** an overall parametric template for the batch is constructed by modeling the affine transformations that may exist among the corresponding populations across samples, and simultaneously
**(2b)** every sample is modeled with its own mixture of skewed and heavy-tailed multivariate probability distributions, which characterizes the high-dimensional populations while registering them using the batch template.
3. Output files are produced containing the fitted models for the batch template and all samples – in formats suitable for visualization and downstream analysis programs. Overlay plots are produced for visual comparison of all class-templates.

There are two options for constructing the parametric models with JCM: the default using mixtures of multivariate skew *t*-distributions and its symmetric counterpart using a mixture of multivariate *t*-distributions.

### Mixtures of multivariate *t*- and skew *t*-distributions

A two-level model is fitted to an input batch or class *C* of *m* samples where each sample is represented by its own 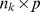 expression matrix, where *k* indexes the sample 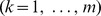. The problem is to simultaneously (a) model all *m* samples in a batch while (b) creating a *p*-dimensional template of *g* components for matching the corresponding populations across all samples. Below we describe the JCM model, for both symmetric and asymmetric components, which are fitted with the JCM-specific EM algorithm for maximum likelihood (ML) estimation as described in detail in Appendices S1 and S2.

Let 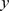 denote a *p*-dimensional vector denoting the values of the *p* markers in a sample. Then JCM provides a method for constructing a template density of *y* for a class of *m* samples, where we let 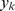 denote the data observed in the *k*th sample (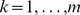). For the construction of the template density, we use a mixture of *g* component distributions, where the latter are members of the *t*-family of distributions [28] or of a skew-extension of this family [7]. In order to define these component distributions, we consider first the *g*-component normal mixture density, which can be expressed as
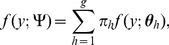
(1)where 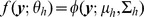 and 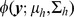 denotes the *p*-variate normal density with mean 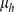 and covariance matrix 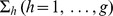; 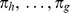 denote the mixing proportions which are non-negative and sum to one. The optimal value of *g* can be specified directly by the user. Alternatively, it can be determined in an unsupervised msanner by the Bayesian Information Criterion (BIC); see Text S2. The vector 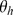 denotes the elements of 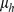 and the elements of 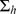 known a priori to be distinct. The vector of unknown parameters is given by 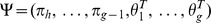, where the superscript *T* denotes vector transpose. In (1), *f* is being used generically to denote a density function.

In the present context where the tails of the normal distribution are heavier or the parameter estimates are affected by atypical observations (outliers), the fitting of mixtures of multivariate *t*-distributions provides a more robust approach to the fitting of normal mixture models [28]. The *t*-component density with location parameter 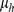, positive-definite scale matrix 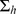, and 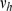 degrees of freedom is given by
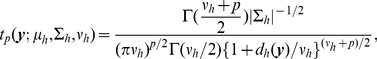
(2)where 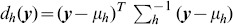 denotes the Mahalanobis squared distance between 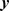 and 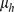 (with 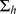 as the scale matrix), and 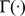 denotes the Gamma function. The parameter 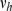 acts as a robustness tuning parameter, which can be inferred from the data by computing its maximum likelihood estimate.

In order to reliably model the clusters that are not elliptically symmetric but are skewed, we shall adopt component densities that are a skewed version of the *t*-distribution. Over the years, a number of proposals have been put forward with increasing level of generality for a skew form of the *t*-distribution. We shall adopt the version proposed by Sahu et al. [29], which is quite general. Accordingly, we let 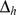 be a diagonal matrix with diagonal elements given by the vector 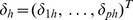 of skewness parameters. Suppose that conditional on a gamma random variable *w* and membership of the *h*th component, the joint distribution of the random vectors 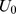 and 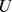 is given by
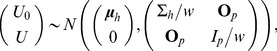
(3)where *w* is distributed according to the gamma 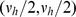 distribution. In the above, we let 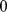 denotes the *p*-dimensional null vector, 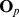 denotes the 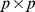 null matrix, and 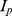 denotes the 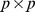 identity matrix.

Then
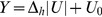
(4)defines a *p*-dimensional multivariate skew *t*-distribution with location 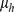, scale matrix 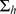, skew (diagonal) matrix 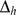, and 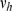 degrees of freedom. Here 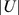 denotes the vector whose *i*th element is equal to the magnitude of the *i*th element of the vector 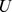. The density of 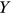 can be expressed as
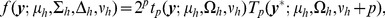
(5)where 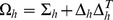, 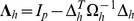, 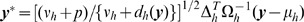. In (5), 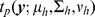 denotes the *p*-variate *t*-density with location 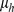, scale matrix 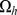, and degrees of freedom 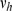, and *T_p_* denotes its (*p*-variate) distribution function.

### Multi-level modeling

We represented the class template by fitting the *g*-component mixture model in (1) to all the *m* samples considered simultaneously, using (2) to represent the *t*-component densities in the symmetric case and (5) in the case of skewed *t*-component densities. If there were no inter-sample variation, then we could proceed to fit the same *t*- or skew *t*-mixture sss to all the *m* samples observed. But here the second-level of JCM model allows for inter-sample variation based on the concept of random-effects, which is often used for combining data from batches containing different amounts of variation. We propose to do so by introducing random-effects terms and using them to specify how the sample-specific component distributions vary from those in the *t*- or skew *t*-mixture model representing the template.

Let *y_ijk_* denote the measurement on the *i*th variable for the *j*th observation in the *k*th sample (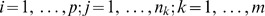). Then conditional on its membership of the *h*th component of the mixture model and conditional on the random-effects terms, we specify the distribution of *y_ijk_* as
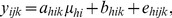
(6)where *e_hijk_* is the error term and where *a_hik_* and *b_hik_* are random-effects terms with
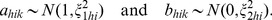
(7)Here 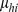 is the *h*th component mean of the *i*th variable in the *g*-component mixture model representing the template for class *C*. The terms *e_hijk_*, *a_hik_* and *b_hik_* are taken to be independent and this independence assumption extends over all variables and all samples. The sample-specific terms, *a_hik_* and *b_hik_*, allow for scaling and translation, respectively, of the sample-component means from the component-means of the template. Estimation of the random-effects model (6) can be performed using the JCM-specific implementation of the EM algorithm described in detail in Appendices S1 and S2.

## Supporting Information

Figure S1 **The workflow of JCM.**
(TIF)

Figure S2 **Spatio-temporal characterization of populations using JCM class templates.**
(TIF)

Figure S3 **Overlay plot for capturing variation within a class.**
(TIF)

Figure S4 **Spatio-temporal profiling of populations representing naïve and memory T cells.**
(TIF)

Figure S5 **Enrichment of cross-panel meta-features.**
(TIF)

Figure S6 **Differences in mound skewness.**
(TIF)

Figure S7 **Running time analysis of JCM.**
(TIF)

Table S1 **The** ***F*** **-measure values of various methods on DLBCL data.**
(PDF)

Appendix S1 **The JCM-MT Model.**
(PDF)

Appendix S2 **The JCM-MST Model.**
(PDF)

Text S1 **Data and experiments.**
(PDF)

Text S2 **The JCM workflow.**
(PDF)
