# Supplementary figures and images for: Joint Modeling and Registration of Cell Populations in Cohorts of High-Dimensional Flow Cytometric Data
Source: PLoS One. 2014 Jul 1;9(7):e100334. doi: 10.1371/journal.pone.0100334 (PMC4077578; doi:10.1371/journal.pone.0100334)

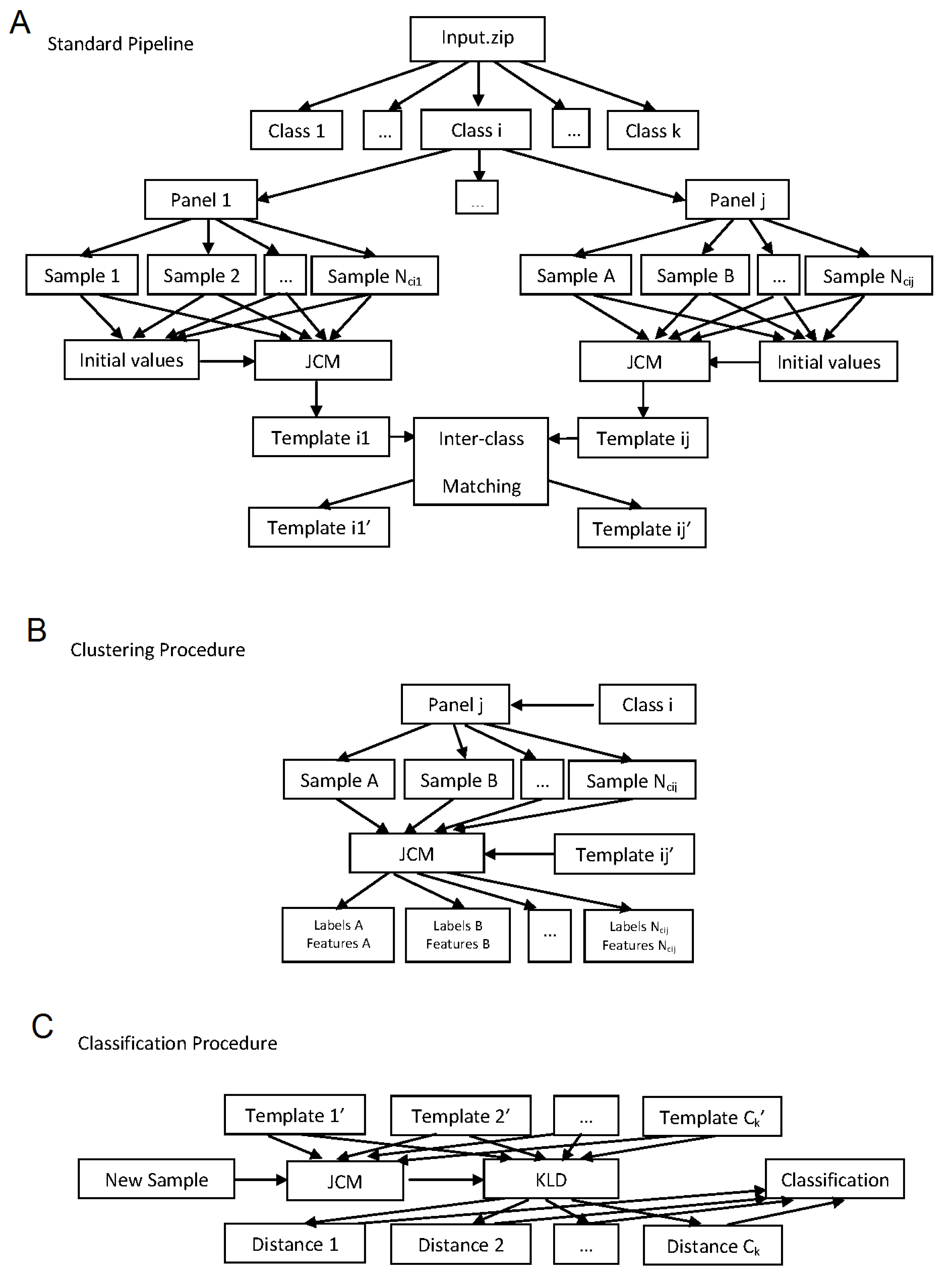

Supplement: Figure S1 — The workflow of JCM. (TIF) [file pone.0100334.s001.tif]

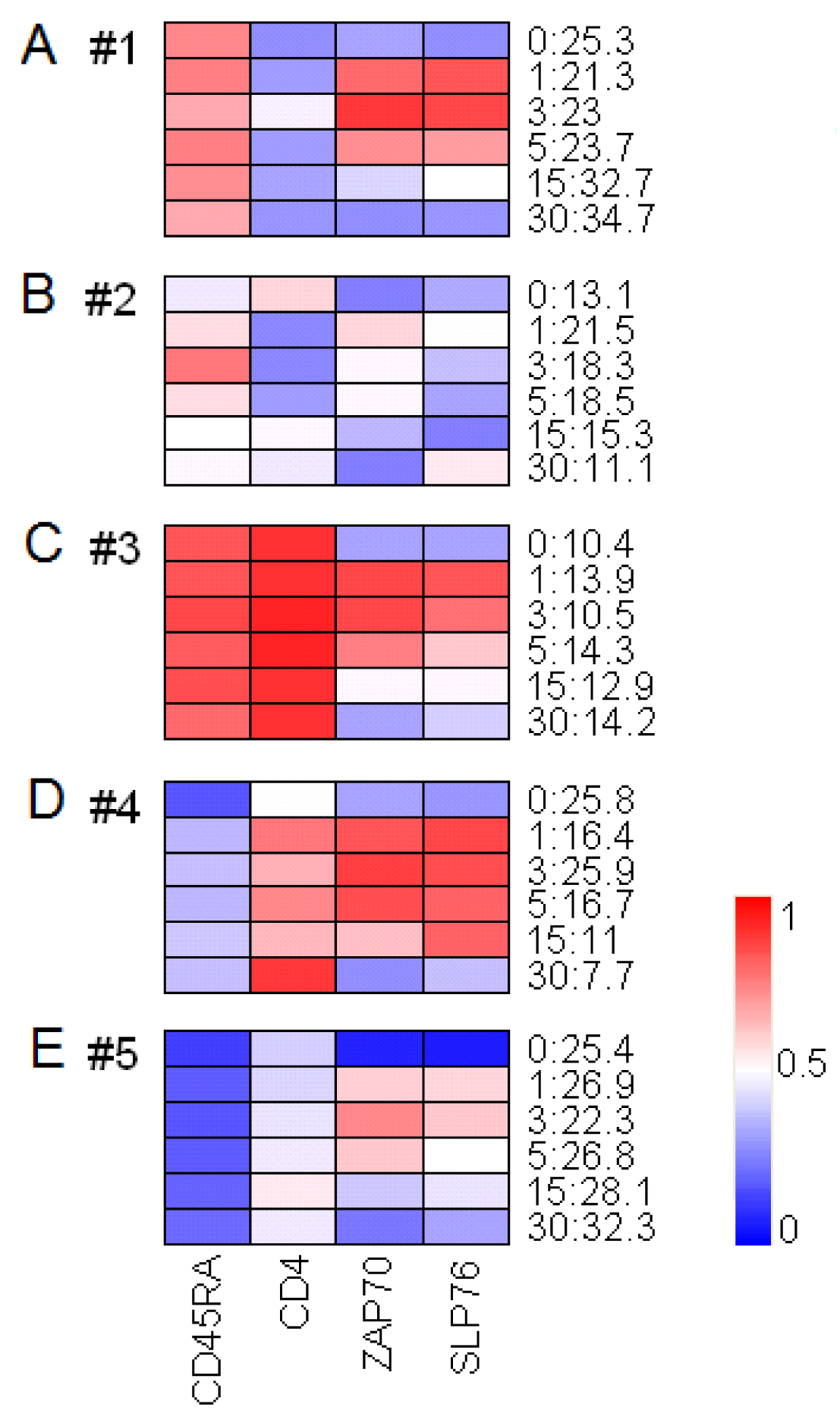

Supplement: Figure S2 — Spatio-temporal characterization of populations using JCM class templates. (TIF) [file pone.0100334.s002.tif]

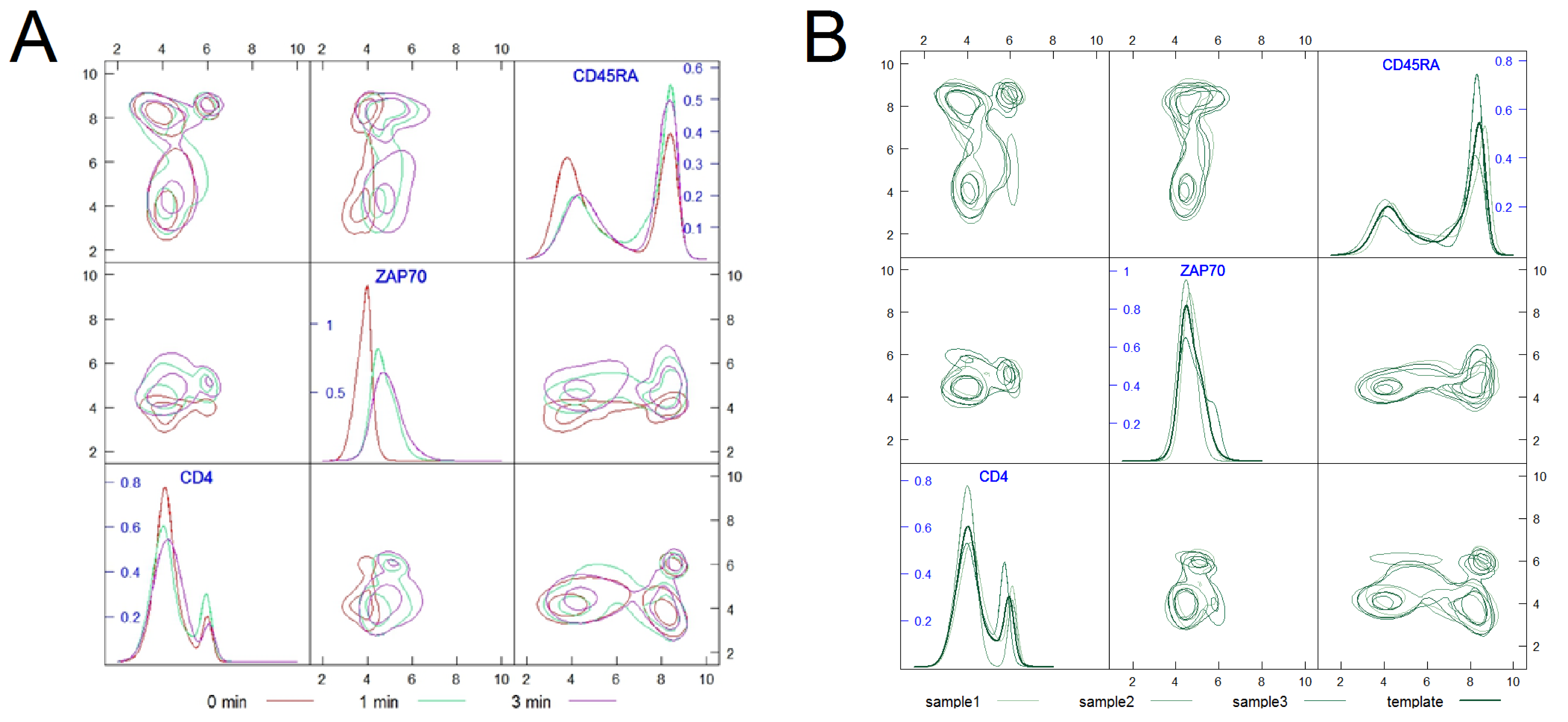

Supplement: Figure S3 — Overlay plot for capturing variation within a class. (TIF) [file pone.0100334.s003.tif]

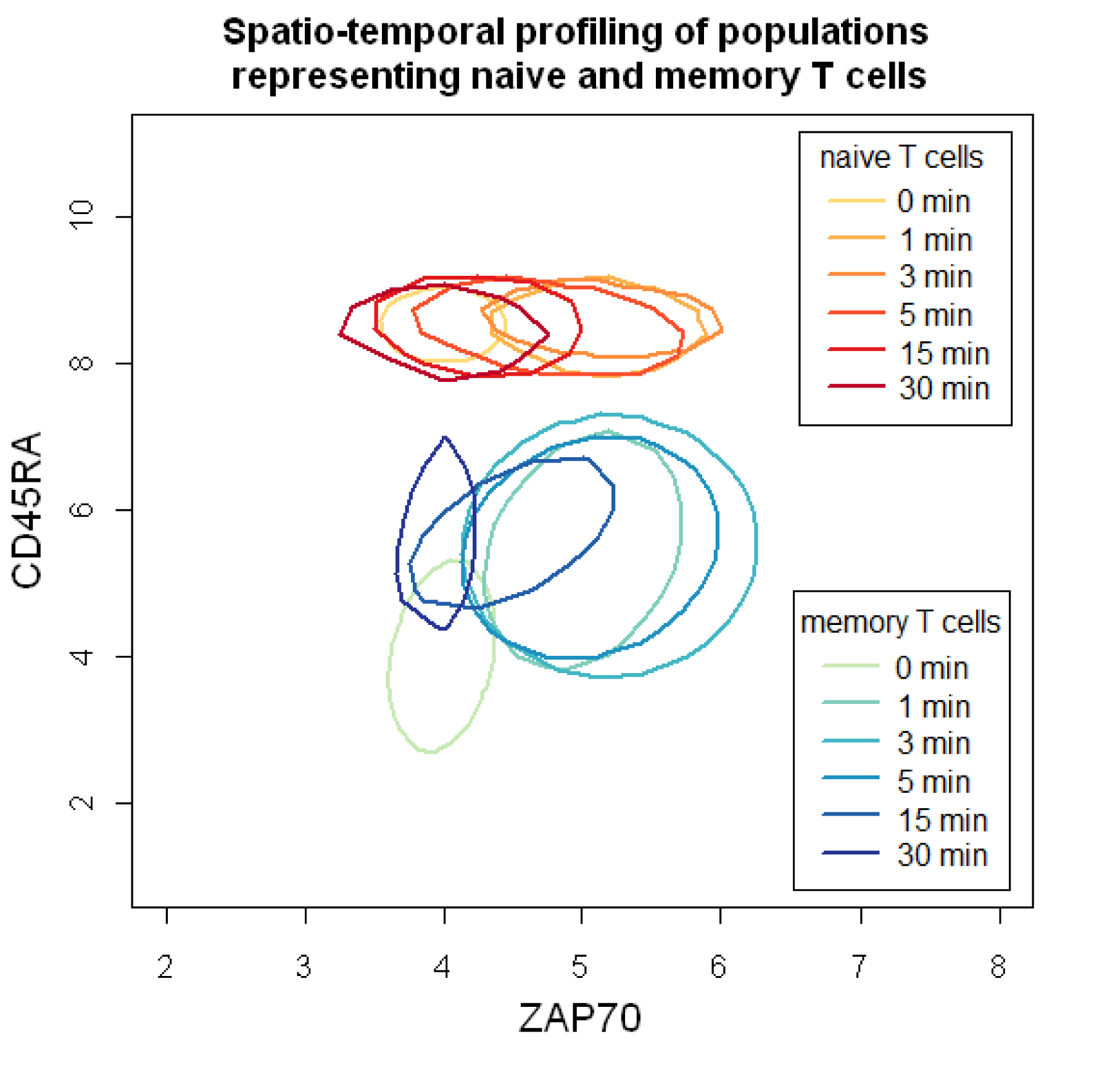

Supplement: Figure S4 — Spatio-temporal profiling of populations representing naïve and memory T cells. (TIF) [file pone.0100334.s004.tif]

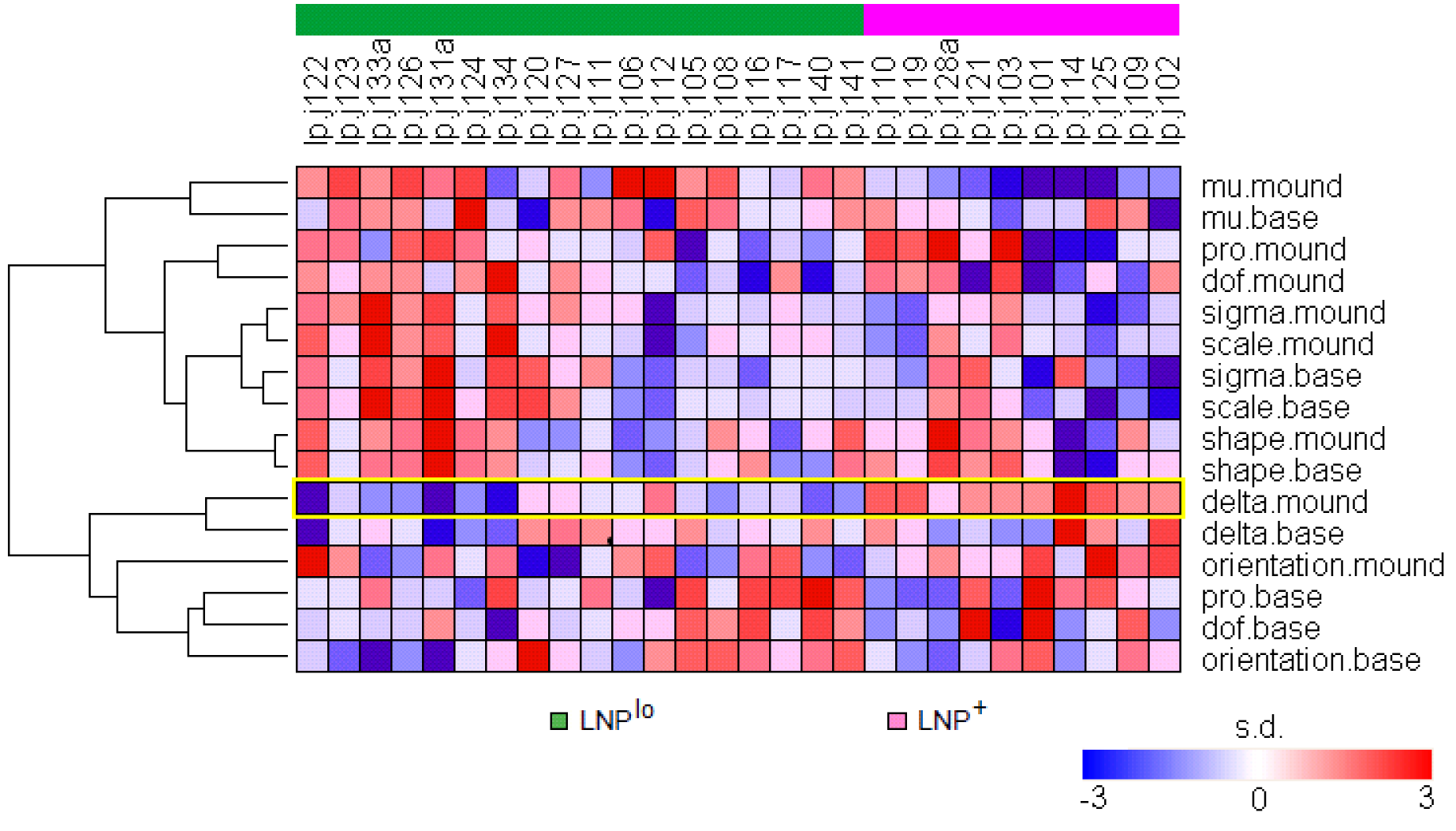

Supplement: Figure S5 — Enrichment of cross-panel meta-features. (TIF) [file pone.0100334.s005.tif]

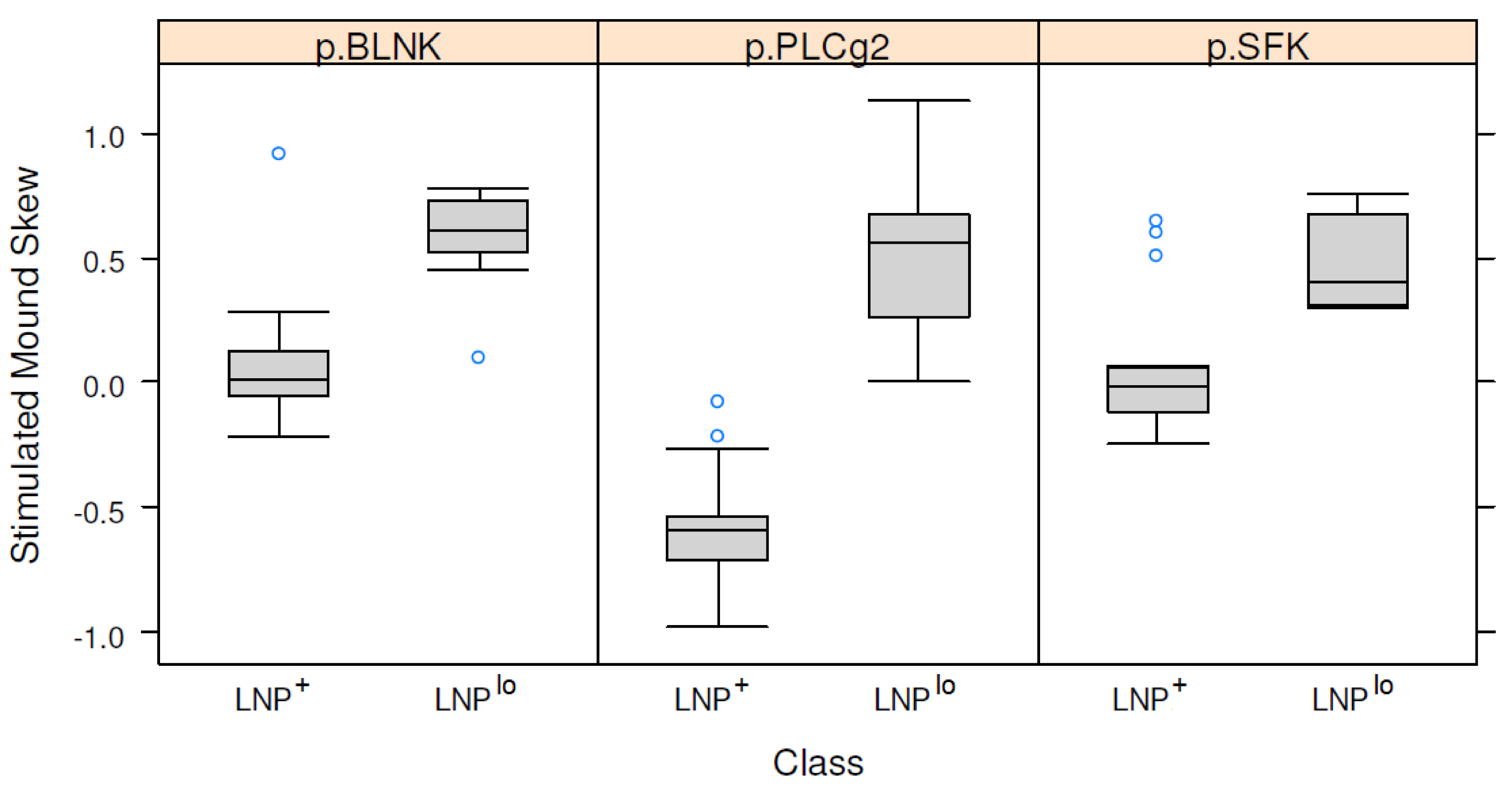

Supplement: Figure S6 — Differences in mound skewness. (TIF) [file pone.0100334.s006.tif]

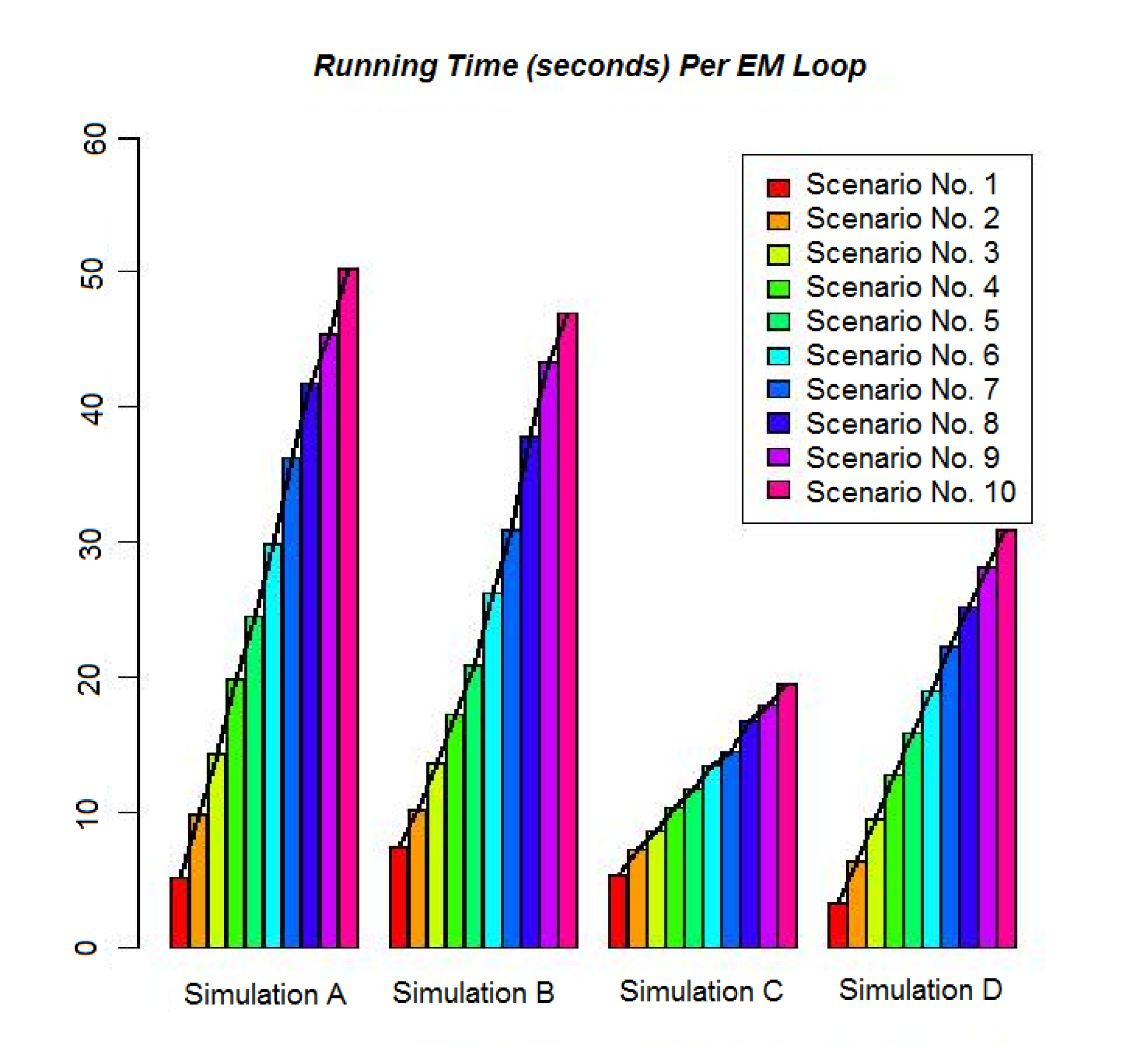

Supplement: Figure S7 — Running time analysis of JCM. (TIF) [file pone.0100334.s007.tif]
