# Supplementary material for: Joint Modeling and Registration of Cell Populations in Cohorts of High-Dimensional Flow Cytometric Data
Source: PLoS One. 2014 Jul 1;9(7):e100334. doi: 10.1371/journal.pone.0100334 (PMC4077578; doi:10.1371/journal.pone.0100334)
Supplement: Table S1 — The F -measure values of various methods on DLBCL data. (PDF) [file pone.0100334.s008.pdf]

Table S1. The F-measure values of various methods on DLBCL data

Sample	JCM	HDPGMM	FLAME	FLAME-I	flowClust-I	SWIFT-I	FLAME-P	flowClust-P	SWIFT-P
Sa001	0.7461	0.8035	0.4784	0.7462	0.7451	0.5035	0.8413	0.8065	0.7455
Sa002	0.9698	0.8786	0.6137	0.9682	0.9665	0.8595	0.8462	0.6952	0.7923
Sa003	0.9408	0.8894	0.4639	0.9363	0.9497	0.5704	0.8508	0.7545	0.8342
Sa004	0.9370	0.8570	0.5090	0.9323	0.9389	0.4994	0.8681	0.8347	0.7591
Sa005	0.9911	0.9609	0.5819	0.8739	0.8678	0.5861	0.8449	0.9242	0.8773
Sa006	0.7802	0.9220	0.4655	0.8017	0.9116	0.6794	0.9196	0.7218	0.8630
Sa007	0.9798	0.9443	0.7603	0.9795	0.9789	0.7582	0.8894	0.9381	0.9543
Sa008	0.9371	0.9042	0.6619	0.9379	0.9146	0.8293	0.9170	0.8933	0.7271
Sa009	0.9818	0.8335	0.7654	0.7958	0.9410	0.7647	0.9623	0.9740	0.9827
Sa010	0.9883	0.6550	0.5104	0.9486	0.9886	0.4992	0.9277	0.9480	0.9312
Sa011	0.9766	0.6049	0.5518	0.9754	0.9753	0.8386	0.8279	0.8493	0.9016
Sa012	0.9904	0.8524	0.7612	0.7250	0.6980	0.7956	0.9548	0.9690	0.9807
Sa013	0.9685	0.6937	0.4553	0.9688	0.9658	0.4478	0.9216	0.9511	0.9433
Sa014	0.9938	0.7209	0.4411	0.9934	0.9917	0.4460	0.9167	0.9843	0.9634
Sa015	0.8936	0.6184	0.5390	0.8942	0.8895	0.4476	0.8934	0.8885	0.9063
Sa016	0.9702	0.6504	0.5130	0.9559	0.8700	0.5676	0.9490	0.9496	0.9393
AF	0.9403	0.7993	0.5670	0.9021	0.9121	0.6308	0.8957	0.8801	0.8813

F-measure for JCM, HDPGMM, FLAME, flowClust and SWIFT on the 16 samples from the DLBCL dataset (see also Tables 1 and 2). For the latter two methods, they were applied to each individual sample separately (denoted with suffix -P). The final row shows the average F-measure (AF) for each method.
