# Supplementary material for: Joint Modeling and Registration of Cell Populations in Cohorts of High-Dimensional Flow Cytometric Data
Source: PLoS One. 2014 Jul 1;9(7):e100334. doi: 10.1371/journal.pone.0100334 (PMC4077578; doi:10.1371/journal.pone.0100334)
Supplement: Appendix S1 — The JCM-MT Model. (PDF) [file pone.0100334.s009.pdf]

Appendix S1 The JCM-MT Model

We describe in this appendix the EM algorithm for the model described in *Methods* with the family of multivariate t-densities as the component distributions. The description is presented for a given patient $k\,(k = 1, \ldots, m)$. For convenience of notation, we shall suppress the subscript k from the random-effects terms a_{hijk} and b_{hjk} as specified by (S2) in Text S2. We let $\boldsymbol{\Psi}_h$ be the diagonal matrix with ith diagonal element equal to $\xi^2_{1hi}\,(i = 1, \ldots, p)$ for $h = 1, \ldots, g$.

1.1 E-step

The EM algorithm is applied iteratively with the E- and M-steps alternate repeatedly. On the $(r + 1)$th iteration, the E-step requires the calculation of the complete-data log likelihood given the observed data, using the current estimate of the parameter vector. This requires the following conditional expectations to be evaluated,

$$
\begin{aligned}
z_{hj}^{(r)} &= E_{\boldsymbol{\Psi}^{(r)}}(z_{hj} = 1 \mid \boldsymbol{y}_j), && \text{(S3)}\\
u_{hj}^{(r)} &= E_{\boldsymbol{\Psi}^{(r)}}(w_{hj} \mid \boldsymbol{y}_j, z_{hj} = 1), && \text{(S4)}\\
\boldsymbol{S}_{1,hj}^{(r)} &= E_{\boldsymbol{\Psi}^{(r)}}(w_{hj}(\boldsymbol{a}_{hj} - \mathbf{1}_p)(\boldsymbol{a}_{hj} - \mathbf{1}_p)^T \mid \boldsymbol{y}_j, z_{hj} = 1), && \text{(S5)}\\
S_{2,hj}^{(r)} &= E_{\boldsymbol{\Psi}^{(r)}}(w_{hj} b_{hj}^2 \mid \boldsymbol{y}_j, z_{hj} = 1), && \text{(S6)}\\
\boldsymbol{S}_{3,hj}^{(r)} &= E_{\boldsymbol{\Psi}^{(r)}}(w_{hj}(\boldsymbol{y}_j - \boldsymbol{\zeta}_h^{(r)}\boldsymbol{a}_{hj} - \mathbf{1}_p b_{hj})(\boldsymbol{y}_j - \boldsymbol{\zeta}_h^{(r)}\boldsymbol{a}_{hj} - \mathbf{1}_p b_{hj})^T \mid \boldsymbol{y}_j, z_{hj} = 1), && \text{(S7)}
\end{aligned}
$$

where

$$\boldsymbol{\zeta}_h^{(r)} = \mathrm{DIAG}(\boldsymbol{\mu}_h^{(r)}).$$

We use $\mathrm{DIAG}(\boldsymbol{a})$ to denote the $p \times p$ diagonal matrix which has diagonal elements given by the p-dimensional vector $\boldsymbol{a}$; we use $\mathrm{diag}(\boldsymbol{A})$ to denote vector given by the diagonal elements of the matrix $\boldsymbol{A}$. For brevity of notation, we suppress the fact that $z_{hj} = 1$ in our equations in the sequel.

It is easy to show that the first two conditional expectations are given by

$$z_{hj}^{(r)} = \frac{\pi_h^{(r)} t_p(\boldsymbol{y}_j; \boldsymbol{\mu}_h^{(r)}, \boldsymbol{\Omega}_h^{(r)}, \nu_h^{(r)})}{\sum_{h=1}^{g} \pi_h^{(r)} t_p(\boldsymbol{y}_j; \boldsymbol{\mu}_h^{(r)}, \boldsymbol{\Omega}_h^{(r)}, \nu_h^{(r)})}, \tag{S8}$$

and

$$u_{hj}^{(r)} = \frac{\nu_h^{(r)} + p}{\nu_h^{(r)} + d_h^{(r)}(\boldsymbol{y}_j)}, \tag{S9}$$

where

$$\boldsymbol{\Omega}_h^{(r)} = \boldsymbol{\zeta}_h^{(r)} \boldsymbol{\Psi}_h^{(r)} \boldsymbol{\zeta}_h^{(r)^T} + \xi_{2h}^{(r)^2} \mathbf{1}_p \mathbf{1}_p^T + \boldsymbol{\Sigma}_h^{(r)}$$

and

$$d_h^{(r)}(\boldsymbol{y}_j) = (\boldsymbol{y}_j - \boldsymbol{\mu}_h^{(r)})^T \boldsymbol{\Omega}_h^{(r)^{-1}} (\boldsymbol{y}_j - \boldsymbol{\mu}_h^{(r)}).$$

To obtain the remaining four conditional expectations, we use the following distributional result,

$$\begin{bmatrix} \boldsymbol{a}_{hj} \\ b_{hj} \\ \boldsymbol{y}_j \end{bmatrix} \mid w_{hj} \sim \mathrm{N}_{2p+1}\left(\begin{bmatrix} \mathbf{1}_p \\ 0 \\ \boldsymbol{\mu}_h \end{bmatrix}, \begin{bmatrix} \boldsymbol{\Psi}_h & \mathbf{0}_p & \boldsymbol{\Psi}_h \boldsymbol{\zeta}_h^T \\ \mathbf{0}_p^T & \xi_{2h}^2 & \xi_{2h}^2 \mathbf{1}_p^T \\ \boldsymbol{\zeta}_h \boldsymbol{\Psi}_h & \xi_{2h}^2 \mathbf{1}_p & \boldsymbol{\Omega}_h \end{bmatrix} \frac{1}{w_{hj}} \right). \tag{S10}$$

Conditional on $\boldsymbol{y}_j$ and w_{hj}, it follows that $[\boldsymbol{a}_{hj} b_{hj}]^T$ has covariance matrix given by

$$\begin{aligned}\boldsymbol{V}_{1,hj}^{(r)} &= \operatorname{cov}_{\boldsymbol{\Psi}^{(r)}}\left(\begin{bmatrix}\boldsymbol{a}_{hj}\\ b_{hj}\end{bmatrix} \mid \boldsymbol{y}_j, w_{hj}\right)\\ &= \frac{1}{w_{hj}}\begin{bmatrix}\boldsymbol{\Psi}_h^{(r)} & 0\\ 0 & \xi_h^{(r)^2}\end{bmatrix} - \frac{1}{w_{hj}}\begin{bmatrix}\boldsymbol{\Psi}_h^{(r)}\boldsymbol{\zeta}_h^{(r)^T}\\ \xi_h^{(r)^2}\mathbf{1}_p^T\end{bmatrix}\boldsymbol{\Omega}_h^{(r)}\left[\boldsymbol{\zeta}_h^{(r)}\boldsymbol{\Psi}_h^{(r)} \;\; \xi_h^{(r)^2}\mathbf{1}_p\right].\end{aligned} \tag{S11}$$

From (11), we have that

$$E_{\boldsymbol{\Psi}^{(r)}}\left(\boldsymbol{a}_{hj} - \mathbf{1}_p \mid \boldsymbol{y}_j, w_{hj}\right) = \boldsymbol{\Psi}_h^{(r)}\boldsymbol{\zeta}_h^{(r)^T}\boldsymbol{\Omega}_h^{(r)^{-1}}\left(\boldsymbol{y}_j - \boldsymbol{\mu}_h^{(r)}\right), \tag{S12}$$

$$E_{\boldsymbol{\Psi}^{(r)}}(b_{hj} \mid \boldsymbol{y}_j, w_{hj}) = \xi_{2h}^{(r)^2}\mathbf{1}_p^T\boldsymbol{\Omega}_h^{(r)^{-1}}\left(\boldsymbol{y}_j - \boldsymbol{\mu}_h^{(r)}\right), \tag{S13}$$

$$\operatorname{cov}_{\boldsymbol{\Psi}^{(r)}}\left(\boldsymbol{a}_{hj} - \mathbf{1}_p \mid \boldsymbol{y}_j, w_{hj}\right) = \frac{1}{w_{hj}}\left(\boldsymbol{\Psi}_h^{(r)} - \boldsymbol{\Psi}_h^{(r)}\boldsymbol{\zeta}_h^{(r)}\boldsymbol{\Omega}_h^{(r)^{-1}}\boldsymbol{\zeta}_h^{(r)}\boldsymbol{\Psi}_h^{(r)}\right), \tag{S14}$$

$$\operatorname{cov}_{\boldsymbol{\Psi}^{(r)}}(b_{hj} \mid \boldsymbol{y}_j, w_{hj}) = \frac{1}{w_{hj}}\left(\xi_{2h}^{(r)^2} - \xi_{2h}^{(r)^2}\mathbf{1}_p^T\boldsymbol{\Omega}_h^{(r)^{-1}}\mathbf{1}_p\right), \tag{S15}$$

$$\operatorname{cov}_{\boldsymbol{\Psi}^{(r)}}\left(\boldsymbol{a}_{hj} b_{hj} \mid \boldsymbol{y}_j, w_{hj}\right) = -\frac{1}{w_{hj}}\left(\xi_{2h}^{(r)^2}\boldsymbol{\Psi}_h^{(r)}\boldsymbol{\zeta}_h^{(r)^T}\boldsymbol{\Omega}_h^{(r)^{-1}}\mathbf{1}_p\right). \tag{S16}$$

By noting that $E(\boldsymbol{X}\boldsymbol{X}^T) = \operatorname{cov}(\boldsymbol{X}) + E(\boldsymbol{X})E(\boldsymbol{X})^T$, the conditional expectations (5), (6), and (7) are given by

$$\boldsymbol{S}_{1,hj}^{(r)} = \boldsymbol{\Psi}_h^{(r)} - \boldsymbol{\Psi}_{ah}^{(r)}\boldsymbol{\zeta}_h^{(r)^T}\boldsymbol{V}_{2,hj}^{(r)}\boldsymbol{\zeta}_h^{(r)}\boldsymbol{\Psi}_h^{(r)}, \tag{S17}$$

$$\boldsymbol{S}_{2,hj}^{(r)} = \xi_{2h}^{(r)^2} - \xi_{2h}^{(r)^2}\mathbf{1}_p^T\boldsymbol{V}_{2,hj}^{(r)}\mathbf{1}_p, \tag{S18}$$

where

$$\boldsymbol{V}_{2,hj}^{(r)} = \left(\boldsymbol{\Omega}_h^{(r)^{-1}} - u_{hj}^{(r)}\boldsymbol{\Omega}_h^{(r)^{-1}}\left(\boldsymbol{y}_j - \boldsymbol{\mu}_h^{(r)}\right)\left(\boldsymbol{y}_j - \boldsymbol{\mu}_h^{(r)}\right)^T\boldsymbol{\Omega}_h^{(r)^{-1}}\right) \tag{S19}$$

and

$$\begin{aligned}\boldsymbol{S}_{3,hj}^{(r)} &= E_{\boldsymbol{\Psi}^{(r)}}\left(w_{hj}\left[\boldsymbol{\zeta}_h^{(r)} \;\; \mathbf{1}_p\right]\boldsymbol{V}_{1,hj}^{(r)}\begin{bmatrix}\boldsymbol{\zeta}_h^{(r)^T}\\ \mathbf{1}_p^T\end{bmatrix} \mid \boldsymbol{y}_j\right) + u_{hj}^{(r)}\boldsymbol{V}_{3,hj}^{(r)}\boldsymbol{V}_{3,hj}^{(r)^T}\\ &= \boldsymbol{V}_{4,hj}^{(r)} - \boldsymbol{V}_{4,hj}^{(r)}\boldsymbol{\Omega}_h^{(r)^{-1}}\boldsymbol{V}_{4,hj}^{(r)} + u_{hj}^{(r)}\boldsymbol{V}_{3,hj}^{(r)}\boldsymbol{V}_{3,hj}^{(r)^T},\end{aligned} \tag{S20}$$

where

$$\boldsymbol{V}_{3,hj}^{(r)} = \boldsymbol{y}_j - \boldsymbol{\mu}_h^{(r)} - \left(\boldsymbol{\zeta}_h^{(r)}\boldsymbol{\Psi}_h^{(r)} + \xi_{2h}^{(r)^2}\mathbf{1}_p\right)\boldsymbol{\Omega}_h^{(r)^{-1}}\left(\boldsymbol{y}_j - \boldsymbol{\mu}_h^{(r)}\right) \tag{S21}$$

and

$$\boldsymbol{V}_{4,hj}^{(r)} = \boldsymbol{\zeta}_h^{(r)}\boldsymbol{\Psi}_h\boldsymbol{\zeta}_h^{(r)} + \xi_{2h}^{(r)^2}\mathbf{1}_p\mathbf{1}_p^T. \tag{S22}$$

In order to calculate $\boldsymbol{\mu}_h^{(r+1)}$, we need to calculate two additional quantities,

$$\boldsymbol{S}_{4,hj}^{(r)} = E_{\boldsymbol{\Psi}^{(r)}}\left(w_{hj}E_{\boldsymbol{\Psi}^{(r)}}\left(\boldsymbol{A}_{hj}\boldsymbol{\Sigma}_h^{(r)^{-1}}\boldsymbol{A}_{hj} \mid \boldsymbol{y}_j, w_{hj}\right) \mid \boldsymbol{y}_j\right) \tag{S23}$$

and

$$\boldsymbol{S}_{5,hj}^{(r)} = E_{\boldsymbol{\Psi}^{(r)}}\left(w_{hj}E_{\boldsymbol{\Psi}^{(r)}}\left(\boldsymbol{A}_{hj}\boldsymbol{\Sigma}_h^{(r)^{-1}}\left(\boldsymbol{y}_j - \boldsymbol{1}_p b_{hj}\right) \mid \boldsymbol{y}_j, w_{hj}\right) \mid \boldsymbol{y}_j\right), \tag{S24}$$

where $\boldsymbol{A}_{hj}$ denotes that diagonal matrix with $\boldsymbol{a}_{hj}$ as its diagonal elements.

Note that

$$E_{\boldsymbol{\Psi}}^{(r)}\left(\boldsymbol{A}_{hj}\boldsymbol{\Sigma}_h^{(r)^{-1}}\boldsymbol{A}_{hj} \mid \boldsymbol{y}_j, w_{hj}\right) = E_{\boldsymbol{\Psi}}^{(r)}\left(\boldsymbol{a}_{hj}\boldsymbol{a}_{hj}^T \mid \boldsymbol{y}_j, w_{hj}\right) \odot \boldsymbol{\Sigma}_h^{(r)^{-1}}, \tag{S25}$$

where $\odot$ denotes the elementwise matrix multiplication. Observe that

$$\begin{aligned} E_{\boldsymbol{\Psi}^{(r)}}\left(\boldsymbol{a}_{hj}\boldsymbol{a}_{hj}^T \mid \boldsymbol{y}_j, w_{hj}\right) &= \frac{1}{w_{hj}}\left(\boldsymbol{\Psi}_h^{(r)} - \boldsymbol{\Psi}_h^{(r)}\boldsymbol{\zeta}_h^{(r)^T}\boldsymbol{\Omega}_h^{(r)^{-1}}\boldsymbol{\zeta}_h^{(r)}\boldsymbol{\Psi}_h^{(r)}\right) \\ &\quad + \left[\boldsymbol{1}_p + \boldsymbol{\Psi}_h^{(r)}\boldsymbol{\zeta}_h^{(r)^T}\boldsymbol{\Omega}_h^{(r)^{-1}}\left(\boldsymbol{y}_j - \boldsymbol{\mu}_h^{(r)}\right)\right] \\ &\quad \times \left[\boldsymbol{1}_p^T + \left(\boldsymbol{y}_j - \boldsymbol{\mu}_h^{(r)}\right)^T\boldsymbol{\Omega}_h^{(r)^{-1}}\boldsymbol{\zeta}_h^{(r)}\boldsymbol{\Psi}_h^{(r)}\right]. \end{aligned} \tag{S26}$$

It follows that

$$\boldsymbol{S}_{4,hj}^{(r)} = \boldsymbol{V}_{5,hj}^{(r)} \odot \boldsymbol{\Sigma}_h^{(r)^{-1}}, \tag{S27}$$

where

$$\begin{aligned} \boldsymbol{V}_{5,hj}^{(r)} &= \left(\boldsymbol{\Psi}_h^{(r)} - \boldsymbol{\Psi}_h^{(r)}\boldsymbol{\zeta}_h^{(r)}\boldsymbol{\Omega}_h^{(r)^{-1}}\boldsymbol{\zeta}_h^{(r)}\boldsymbol{\Psi}_h^{(r)}\right) \\ &\quad + u_{hj}^{(r)}\left[\boldsymbol{1}_p + \boldsymbol{\Psi}_h^{(r)}\boldsymbol{\zeta}_h^{(r)}\boldsymbol{\Omega}_h^{(r)^{-1}}\left(\boldsymbol{y}_j - \boldsymbol{\mu}_h^{(r)}\right)\right] \\ &\quad \times \left[\boldsymbol{1}_p + \boldsymbol{\Psi}_h^{(r)}\boldsymbol{\zeta}_h^{(r)}\boldsymbol{\Omega}_h^{(r)^{-1}}\left(\boldsymbol{y}_j - \boldsymbol{\mu}_h^{(r)}\right)\right]^T. \end{aligned} \tag{S28}$$

To calculate $\boldsymbol{S}_{5,hj}^{(r)}$, we note that

$$\begin{aligned} &= \boldsymbol{E}_{\boldsymbol{\Psi}^{(r)}}\left(\boldsymbol{A}_{hj}\boldsymbol{\Sigma}_h^{(r)^{-1}}\left(\boldsymbol{y}_j - \boldsymbol{1}_p b_{hj}\right) \mid \boldsymbol{y}_j, w_{hj}\right) \\ &= E_{\boldsymbol{\Psi}}^{(r)}\left(\boldsymbol{A}_{hj} \mid \boldsymbol{y}_j, w_{hj}\right)\boldsymbol{\Sigma}_h^{(r)^{-1}}\boldsymbol{y}_j - E_{\boldsymbol{\Psi}^{(r)}}\left(\boldsymbol{A}_{hj}b_{hj} \mid \boldsymbol{y}_j, w_{hj}\right)\boldsymbol{\Sigma}_h^{(r)^{-1}}\boldsymbol{1}_p. \end{aligned}$$

Then

$$\begin{aligned} \boldsymbol{S}_{5,hj}^{(r)} &= u_{hj}^{(r)}\boldsymbol{V}_{6,hj}^{(r)}\boldsymbol{\Sigma}_h^{(r)^{-1}}\boldsymbol{y}_j + \text{ DIAG}\left(\xi_{2h}^{(r)^2}\boldsymbol{\Psi}_h^{(r)}\boldsymbol{\zeta}_h^{(r)}\boldsymbol{\Omega}_h^{(r)^{-1}}\boldsymbol{1}_p\right)\boldsymbol{\Sigma}_h^{(r)^{-1}}\boldsymbol{1}_p, \\ &\quad - u_{hj}^{(r)}\text{DIAG}\left[\xi_{2h}^{(r)^2}\boldsymbol{V}_{6,hj}^{(r)}\boldsymbol{1}_p^T\boldsymbol{\Omega}_h^{(r)^{-1}}\left(\boldsymbol{y}_j - \boldsymbol{\mu}_h^{(r)}\right)\right]\boldsymbol{\Sigma}_h^{(r)^{-1}}\boldsymbol{1}_p, \end{aligned} \tag{S29}$$

where

$$\boldsymbol{V}_{6,hj}^{(r)} = \text{DIAG}\left(\boldsymbol{1}_p + \boldsymbol{\Psi}_h^{(r)}\boldsymbol{\zeta}_h^{(r)}\boldsymbol{\Omega}_h^{(r)^{-1}}\left(\boldsymbol{y}_j - \boldsymbol{\mu}_h^{(r)}\right)\right). \tag{S30}$$

1.2 M-step

The estimates of the parameters are updated on the M-step by maximizing the Q-function over the parameter space. The Q-function is equal to the conditional expectation of the complete-data log likelihood given the observed data, using the current fit for the vector of unknown parameters. It follows that

$$\begin{aligned}
\pi_h^{(r+1)} &= \frac{1}{n}\sum_{j=1}^{n} z_{hj}^{(r)}, \\
\mu_h^{(r+1)} &= \left(\sum_{j=1}^{n} z_{hj}^{(r)} \boldsymbol{S}_{4,hj}^{(r)}\right)^{-1} \sum_{j=1}^{n} z_{hj}^{(r)} \boldsymbol{S}_{5,hj}^{(r)}, \\
\boldsymbol{\Sigma}_h^{(r+1)} &= \frac{\sum_{j=1}^{n} z_{hj}^{(r)} \boldsymbol{S}_{3,hj}^{(r)}}{\sum_{j=1}^{n} z_{hj}^{(r)}}, \\
\boldsymbol{\Psi}_h^{(r+1)} &= \frac{\sum_{j=1}^{n} z_{hj}^{(r)} \boldsymbol{S}_{1,hj}^{(r)}}{\sum_{j=1}^{n} z_{hj}^{(r)}}, \\
\xi_{2h}^{(k+1)^2} &= \frac{\sum_{j=1}^{n} z_{hj}^{(r)} S_{2,hj}^{(r)}}{\sum_{j=1}^{n} z_{hj}^{(r)}}.
\end{aligned} \tag{S31}$$

The update of the degrees of freedom $\nu_h^{(r+1)}$ is given implicitly as a solution of the equation

$$\frac{\sum_{j=1}^{n} z_{hj}^{(r)} \left[\log(u_{hj}^{(r)}) - u_{hj}^{(r)} - \log\left(\frac{\nu_h^{(r)}+p}{2}\right) + \psi\left(\frac{\nu_h^{(r)}+p}{2}\right)\right]}{\sum_{j=1}^{n} z_{hj}^{(r)}} + \log\left(\frac{\nu_h}{2}\right) - \psi\left(\frac{\nu_h}{2}\right) + 1 = 0, \tag{S32}$$

where $\psi(\cdot)$ denotes the Digamma function.
