# Supplementary material for: Joint Modeling and Registration of Cell Populations in Cohorts of High-Dimensional Flow Cytometric Data
Source: PLoS One. 2014 Jul 1;9(7):e100334. doi: 10.1371/journal.pone.0100334 (PMC4077578; doi:10.1371/journal.pone.0100334)
Supplement: Appendix S2 — The JCM-MST Model. (PDF) [file pone.0100334.s010.pdf]

Appendix S2 The JCM-MST Model

In this Appendix, we describe the EM algorithm for estimating the parameters of the model described in *Methods* where the components are multivariate skew t-densities given by (S1) in Text S2.

2.1 E-step

As in Appendix S1, on the E-step of the EM algorithm we have to calculate the conditional expectation of the complete-data log likelihood given the observed data. It requires the following conditional expectations to be calculated,

$$
\begin{aligned}
z_{hj}^{(r)} &= E_{\boldsymbol{\Psi}^{(r)}}\left(z_{hj}=1 \mid \boldsymbol{y}_j\right), && \text{(S33)}\\
u_{hj}^{(r)} &= E_{\boldsymbol{\Psi}^{(r)}}\left(w_{hj} \mid \boldsymbol{y}_j, z_{hj}=1\right), && \text{(S34)}\\
S_{1,hj}^{(r)} &= E_{\boldsymbol{\Psi}^{(r)}}\left(w_{hj}u_{hj} \mid \boldsymbol{y}_j, u_{hj}>0, z_{hj}=1\right), && \text{(S35)}\\
S_{2,hj}^{(r)} &= E_{\boldsymbol{\Psi}^{(r)}}\left(w_{hj}u_{hj}^2 \mid \boldsymbol{y}_j, u_{hj}>0, z_{hj}=1\right), && \text{(S36)}\\
\boldsymbol{S}_{3,hj}^{(r)} &= E_{\boldsymbol{\Psi}^{(r)}}\left(w_{hj}\left(\boldsymbol{a}_{hj}-\mathbf{1}_p\right)\left(\boldsymbol{a}_{hj}-\mathbf{1}_p\right)^T \mid \boldsymbol{y}_j, u_{hj}>0, z_{hj}=1\right), && \text{(S37)}\\
S_{4,hj}^{(r)} &= E_{\boldsymbol{\Psi}^{(r)}}\left(w_{hj}b_{hj}^2 \mid \boldsymbol{y}_j, u_{hj}>0, z_{hj=1}\right), && \text{(S38)}\\
\boldsymbol{S}_{5,hj}^{(r)} &= E_{\boldsymbol{\Psi}^{(r)}}\left(w_{hj}u_{hj}(\boldsymbol{y}_j-\boldsymbol{\zeta}_h\boldsymbol{a}_{hj}-\mathbf{1}_p b_{hj}) \mid \boldsymbol{y}_j, u_{hj}>0, z_{hj}=1\right), && \text{(S39)}\\
\boldsymbol{S}_{6,hj}^{(r)} &= E_{\boldsymbol{\Psi}^{(r)}}\left(w_{hj}\boldsymbol{\epsilon}_{hj}\boldsymbol{\epsilon}_{hj}^T \mid \boldsymbol{y}_j, u_{hj}>0, z_{hj}=1\right), && \text{(S40)}\\
\boldsymbol{S}_{7,hj}^{(r)} &= E_{\boldsymbol{\Psi}^{(r)}}\left(w_{hj}\boldsymbol{A}_{hj}\boldsymbol{\Sigma}_h^{(r)^{-1}}\boldsymbol{A}_{hj} \mid \boldsymbol{y}_j, u_{hj}>0, z_{hj}=1\right), && \text{(S41)}\\
\boldsymbol{S}_{8,hj}^{(r)} &= E_{\boldsymbol{\Psi}^{(r)}}\left(w_{hj}\boldsymbol{A}_{hj}\boldsymbol{\Sigma}_h^{(r)^{-1}}(\boldsymbol{y}_j-\mathbf{1}_p b_{hj}-\boldsymbol{\delta}_h u_{hj}) \mid \boldsymbol{y}_j, u_{hj}>0, z_{hj}=1\right), && \text{(S42)}
\end{aligned}
$$

where $\boldsymbol{\epsilon}_{hj}=(\boldsymbol{y}_j-\boldsymbol{\zeta}_h\boldsymbol{a}_{hj}-\mathbf{1}_p b_{hj}-\boldsymbol{\delta}_h u_{hj})$. For brevity of notation, we suppress the fact that $z_{hj}=1$ in our subsequent equations.

It is easy to show that

$$
z_{hj}^{(r)} = \frac{\pi_h f(\boldsymbol{y}_j; \boldsymbol{\mu}_h^{(r)}, \tilde{\boldsymbol{\Omega}}_h^{(r)}, \boldsymbol{\delta}_h^{(r)}, v_h^{(r)})}{\sum_{h=1}^{g}\pi_h^{(r)} f(\boldsymbol{y}_j; \boldsymbol{\mu}_h^{(r)}, \tilde{\boldsymbol{\Omega}}_h^{(r)}, \boldsymbol{\delta}_h^{(r)}, \nu_h^{(r)})}, \tag{S43}
$$

where $f(\boldsymbol{y}_j; \boldsymbol{\mu}_h^{(r)}, \boldsymbol{\Omega}_h^{(r)}, \boldsymbol{\delta}_h^{(r)}, \nu_h^{(r)})$ is the density function of the skew t-distribution, given by

$$
2t_p\left(\boldsymbol{y}_j; \boldsymbol{\mu}_h^{(r)}, \tilde{\boldsymbol{\Omega}}_h^{(r)}, \nu_h^{(r)}\right) T_1\left(\frac{\xi_{hj}^{(r)}}{\sigma_h^{(r)}}\sqrt{\frac{\nu_h^{(r)}+p}{\nu_h^{(r)}+d_h^{(r)}(\boldsymbol{y}_j)}}; 0, 1, \nu_h^{(r)}+p\right), \tag{S44}
$$

and

$$
\begin{aligned}
\boldsymbol{\zeta}_h^{(r)} &= \mathrm{DIAG}(\boldsymbol{\mu}_h^{(r)}), \\
\boldsymbol{\Omega}_h^{(r)} &= \boldsymbol{\zeta}_h^{(r)} \boldsymbol{\Psi}_h^{(r)} \boldsymbol{\zeta}_h^{(r)^T} + \xi_{2h}^{(r)^2} \mathbf{1}_p \mathbf{1}_p^T + \boldsymbol{\Sigma}_h^{(r)}, \\
\tilde{\boldsymbol{\Omega}}_h^{(r)} &= \boldsymbol{\zeta}_h^{(r)} \boldsymbol{\Psi}_h^{(r)} \boldsymbol{\zeta}_h^{(r)^T} + \xi_{2h}^{(r)^2} \mathbf{1}_p \mathbf{1}_p^T + \boldsymbol{\Sigma}_h^{(r)} + \boldsymbol{\delta}_h \boldsymbol{\delta}_h^T, \\
\xi_{hj}^{(r)} &= \boldsymbol{\delta}_h^{(r)^T} \tilde{\boldsymbol{\Omega}}_h^{(r)} \left(\boldsymbol{y}_j - \boldsymbol{\mu}_h^{(r)} \right), \\
\sigma_h^{(r)^2} &= 1 - \boldsymbol{\delta}_h^{(r)^T} \tilde{\boldsymbol{\Omega}}_h^{(r)^{-1}} \boldsymbol{\delta}_h^{(r)}, \\
d_h^{(r)}(\boldsymbol{y}_j) &= (\boldsymbol{y}_j - \boldsymbol{\mu}_h^{(r)})^T \tilde{\boldsymbol{\Omega}}_h^{(r)^{-1}} (\boldsymbol{y}_j - \boldsymbol{\mu}_h^{(r)}).
\end{aligned}
$$

Concerning the half-normal distribution, we have that the mean and variance of u_{hj} conditional on $\boldsymbol{y}_j$ and w_{hj}, are given by

$$
E_{\boldsymbol{\Psi}^{(r)}}(u_{hj} \mid \boldsymbol{y}_j, w_{hj}) = \xi_{hj}^{(r)}
$$

$$
\mathrm{var}_{\boldsymbol{\Psi}^{(r)}}(u_{hj} \mid \boldsymbol{y}_j, w_{hj}) = \sigma_h^{(r)^2} / w_{hj}.
$$

It follows then that we can obtain the following (conditional) moments of the half-normal distribution,

$$
\begin{aligned}
E_{\boldsymbol{\Psi}^{(r)}}(u_{hj} \mid \boldsymbol{y}_j, w_{hj}, u_{hj} > 0) &= \xi_{hj} + \frac{\sigma_h^{(r)}}{\sqrt{w_{hj}}} \frac{\phi\left(\frac{\xi_{hj}^{(r)}}{\sigma_h^{(r)}} \sqrt{w_{hj}}\right)}{\Phi\left(\frac{\xi_{hj}^{(r)}}{\sigma_h^{(r)}} \sqrt{w_{hj}}\right)}, \\
E_{\boldsymbol{\Psi}^{(r)}}(u_{hj}^2 \mid \boldsymbol{y}_j, w_{hj}, u_{hj} > 0) &= \xi_{hj}^{(r)2} + \frac{\sigma_h^{(r)4}}{w_{hj}} + \frac{\xi_{hj}^{(r)} \sigma_h^{(r)}}{\sqrt{w_{hj}}} \frac{\phi\left(\frac{\xi_{hj}^{(r)}}{\sigma_h^{(r)}} \sqrt{w_{hj}}\right)}{\Phi\left(\frac{\xi_{hj}^{(r)}}{\sigma_h^{(r)}} \sqrt{w_{hj}}\right)},
\end{aligned}
\tag{S45}
$$

where ϕ and Φ denote the standard univariate normal density and (cumulative) distribution function, respectively.

To find the corresponding moments unconditional on w, we need the conditional density function of w given y,

$$
f(w \mid \boldsymbol{y}_j, u_{hj} > 0) = \frac{\Phi\left(\frac{\xi_{hj}^{(r)}}{\sigma_h^{(r)}} \sqrt{w}\right)}{T_1\left(\frac{\xi_{hj}^{(r)}}{\sigma_h^{(r)}} \sqrt{\frac{\nu_h^{(r)} + p}{\nu_h^{(r)} + d_h^{(r)}(\boldsymbol{y}_j)}}; 0, 1, \nu_h^{(r)} + p\right)} \; f_G\left(w; \frac{\nu_h^{(r)} + p}{2}, \frac{\nu_h^{(r)} + d_h^{(r)}(\boldsymbol{y}_j)}{2}\right), \tag{S46}
$$

where $f_G(\cdot; \alpha, \beta)$ denotes the gamma density function with shape and scale parameters given by α and β respectively.

It follows that

$$
\begin{aligned}
w_{hj}^{(r)} &= E_{\boldsymbol{\Psi}^{(r)}}(w_{hj} \mid \boldsymbol{y}_j, u_{hj} > 0, z_{hj} = 1) \\
&= \int_0^\infty \frac{\Phi\left(\frac{\xi_{hj}^{(r)}}{\sigma_h^{(r)}}\sqrt{w}\right)}{T_1\left(\frac{\xi_{hj}^{(r)}}{\sigma_h^{(r)}}\sqrt{\frac{\nu_h^{(r)}+p}{\nu_h^{(r)}+d_h^{(r)}(\boldsymbol{y}_j)}};0,1,\nu_h^{(r)}+p\right)} \frac{\left(\frac{\nu_h^{(r)}+d_h^{(r)}(\boldsymbol{y}_j)}{2}\right)^{\frac{\nu_h^{(r)}+p}{2}}}{\Gamma\left(\frac{\nu_h^{(r)}+p}{2}\right)} w^{\frac{\nu_h^{(r)}+p+2}{2}-1} e^{-\frac{w\left(\nu_h^{(r)}+d_h^{(r)}(\boldsymbol{y}_j)\right)}{2}} dw \\
&= \frac{\nu_h^{(r)}+p}{\nu_h^{(r)}+d_h^{(r)}(\boldsymbol{y}_j)} \frac{T_1\left(\frac{\xi_{hj}^{(r)}}{\sigma_h^{(r)}}\sqrt{\frac{\nu_h^{(r)}+p+2}{\nu_h^{(r)}+d_h^{(r)}(\boldsymbol{y}_j)}};0,1,\nu^{(r)}+p+2\right)}{T_1\left(\frac{\xi_{hj}^{(r)}}{\sigma_h^{(r)}}\sqrt{\frac{\nu_h^{(r)}+p}{\nu_h^{(r)}+d_h^{(r)}(\boldsymbol{y}_j)}};0,1,\nu_h^{(r)}+p\right)}, \qquad \text{(S47)}
\end{aligned}
$$

and

$$
\begin{aligned}
\boldsymbol{V}_{1,hj}^{(r)} &= E_{\boldsymbol{\Psi}^{(r)}}\left(\sqrt{w_{hj}}\frac{\phi\left(\frac{\xi_{hj}(r)}{\sigma_h^{(r)}}\sqrt{w_{hj}}\right)}{\Phi\left(\frac{\xi_{hj}^{(r)}}{\sigma^{(r)}}\sqrt{w_{hj}}\right)} \mid \boldsymbol{y}_j, u_{hj} > 0\right) \\
&= \left[\sqrt{\pi\left(\nu_h^{(r)} + \tilde{d}_h^{(r)}(\boldsymbol{y}_j)\right)} T_1\left(\frac{\xi_{hj}^{(r)}}{\sigma_h^{(r)}}\sqrt{\frac{\nu_h^{(r)}+p}{\nu_h^{(r)}+d_h^{(r)}(\boldsymbol{y}_j)}}\right)\right]^{-1} \left(\frac{\nu_h^{(r)}+d_h^{(r)}(\boldsymbol{y}_j)}{\nu_h+\tilde{d}_h^{(r)}(\boldsymbol{y}_j)}\right)^{\frac{\nu_h^{(r)}+p}{2}} \frac{\Gamma\left(\frac{\nu_h^{(r)}+p+1}{2}\right)}{\Gamma\left(\frac{\nu_h^{(r)}+p}{2}\right)},
\end{aligned}
$$

where $\tilde{d}_h^{(r)}(\boldsymbol{y}_j) = d_h^{(r)}(\boldsymbol{y}_j) + \frac{\xi_{hj}^{(r)^2}}{\sigma_h^2}$.

We are now in a position to calculate the required conditional expectations,

$$
\begin{aligned}
S_{1,hj}^{(r)} &= E_{\boldsymbol{\Psi}^{(r)}}(w_{hj}u_{hj} \mid \boldsymbol{y}_j, u_{hj} > 0) \\
&= w_{hj}^{(r)}\xi_{hj}^{(r)} + \sigma_h^{(r)}\boldsymbol{V}_{1,hj}^{(r)}, \qquad \text{(S48)} \\
S_{2,hj}^{(r)} &= E_{\boldsymbol{\Psi}^{(r)}}\left(w_{hj}u_{hj}^2 \mid \boldsymbol{y}_j, u_{hj} > 0\right), \\
&= w_{hj}^{(r)}\xi_{hj}^{(r)^2} + \sigma_h^{(r)^4} + \sigma_h^{(r)}\xi_{hj}^{(r)}V_{1,hj}^{(r)}. \qquad \text{(S49)}
\end{aligned}
$$

To calculate the remaining conditional expectations, note that

$$
\begin{aligned}
&E_{\boldsymbol{\Psi}^{(r)}}\left(\begin{bmatrix} \boldsymbol{a}_{hj} \\ b_{hj} \end{bmatrix} \mid \boldsymbol{y}_j, w_{hj}, u_{hj}\right) \\
&= \begin{bmatrix} \mathbf{1}_p \\ 0 \end{bmatrix} + \begin{bmatrix} \boldsymbol{\Psi}_h^{(r)}\boldsymbol{\zeta}_h^{(r)} \\ \xi_{2h}^{(r)^2}\mathbf{1}_p^T \end{bmatrix} \boldsymbol{\Omega}_h^{(r)^{-1}}\left(\boldsymbol{y}_j - \boldsymbol{\mu}_h^{(r)} - \boldsymbol{\delta}_h^{(r)}u_{hj}\right) \qquad \text{(S50)}
\end{aligned}
$$

and

$$
\begin{aligned}
&\boldsymbol{V}_{2,hj}^{(r)} = \text{cov}_{\boldsymbol{\Psi}^{(r)}}\left(\begin{bmatrix} \boldsymbol{a}_{hj} \\ b_{hj} \end{bmatrix} \mid \boldsymbol{y}_j, w_{hj}, u_{hj}\right) \\
&= \frac{1}{w_{hj}}\begin{bmatrix} \boldsymbol{\Psi}_h^{(r)} & 0 \\ 0 & \xi_{2h}^{(r)^2} \end{bmatrix} - \frac{1}{w_{hj}}\begin{bmatrix} \boldsymbol{\Psi}_h^{(r)}\boldsymbol{\zeta}_h^{(r)} \\ \xi_{2h}^{(r)^2}\mathbf{1}_p^T \end{bmatrix} \boldsymbol{\Omega}_h^{(r)^{-1}} \begin{bmatrix} \boldsymbol{\zeta}_h^{(r)}\boldsymbol{\Psi}_h^{(r)} & \xi_{2h}^{(r)^2}\mathbf{1}_p \end{bmatrix}. \qquad \text{(S51)}
\end{aligned}
$$

From the above, we have

$$E_{\mathbf{\Psi}^{(r)}}\left(\boldsymbol{a}_{hj}-\mathbf{1}_p \mid \boldsymbol{y}_j, w_{hj}, u_{hj}\right)=\mathbf{\Psi}_h^{(r)}\boldsymbol{\zeta}^{(r)}\mathbf{\Omega}_h^{(r)^{-1}}\left(\boldsymbol{y}_j-\boldsymbol{\mu}_h^{(r)}-\boldsymbol{\delta}_h^{(r)}u_{hj}\right),$$
$$E_{\mathbf{\Psi}^{(r)}}\left(b_{hj} \mid \boldsymbol{y}_j, w_{hj}, u_{hj}\right)=\xi_{2h}^{(r)^2}\mathbf{1}_p^{(r)^T}\mathbf{\Omega}_h^{(r)^{-1}}\left(\boldsymbol{y}_j-\boldsymbol{\mu}_h^{(r)}-\boldsymbol{\delta}_h^{(r)}u_{hj}\right),$$

and

$$\operatorname{cov}_{\mathbf{\Psi}^{(r)}}\left(\boldsymbol{a}_{hj}-\mathbf{1}_p \mid \boldsymbol{y}_j, w_{hj}, u_{hj}\right)=\frac{1}{w_{hj}}\left(\mathbf{\Psi}_h^{(r)}-\mathbf{\Psi}_h^{(r)}\boldsymbol{\zeta}_h^{(r)^T}\mathbf{\Omega}_h^{(r)^{-1}}\boldsymbol{\zeta}_h^{(r)}\mathbf{\Psi}_h^{(r)}\right),$$
$$\operatorname{cov}_{\mathbf{\Psi}^{(r)}}\left(b_{hj} \mid \boldsymbol{y}_j, w_{hj}, u_{hj}\right)=\frac{1}{w_{hj}}\left(\xi_{2h}^{(r)^2}-\xi_{2h}^{(r)^2}\mathbf{1}_p^T\mathbf{\Omega}_h^{(r)^{-1}}\mathbf{1}_p\right),$$
$$\operatorname{cov}_{\mathbf{\Psi}^{(r)}}\left(\boldsymbol{a}_{hj}b_{hj} \mid \boldsymbol{y}_j, w_{hj}, u_{hj}\right)=\frac{1}{w_{hj}}\left(-\xi_{2h}^{(r)^2}\mathbf{\Psi}_h^{(r)}\boldsymbol{\zeta}_h^{(r)}\mathbf{\Omega}_h^{(r)^{-1}}\mathbf{1}_p\right).$$

Also,

$$\begin{aligned}
&\operatorname{cov}_{\mathbf{\Psi}^{(r)}}\left(\boldsymbol{y}_j-\boldsymbol{\zeta}_h^{(r)}\boldsymbol{a}_{hj}-\mathbf{1}_p b_{hj} \mid \boldsymbol{y}_j, w_{hj}, u_{hj}\right)\\
&=\left[\boldsymbol{\zeta}_h^{(r)} \quad \mathbf{1}_p\right]\boldsymbol{V}_{2,hj}^{(r)}\left[\begin{array}{c}\boldsymbol{\zeta}_h^{(r)^T}\\ \mathbf{1}_p^T\end{array}\right]+\boldsymbol{V}_{3,hj}^{(r)}\boldsymbol{V}_{3,hj}^{(r)^T}\\
&=\frac{1}{w_{hj}}\left(\boldsymbol{V}_{4,hj}^{(r)}-\boldsymbol{V}_{4,hj}^{(r)}\mathbf{\Omega}_h^{(r)^{-1}}\boldsymbol{V}_{4,hj}^{(r)}\right)+\boldsymbol{V}_{3,hj}^{(r)}\boldsymbol{V}_{3,hj}^{(r)^T},
\end{aligned}$$

where

$$\boldsymbol{V}_{3,hj}^{(r)}=\boldsymbol{y}_j-\boldsymbol{\mu}_h^{(r)}-\left(\boldsymbol{\zeta}_h^{(r)}\mathbf{\Psi}_h^{(r)}+\xi_h^{(r)^2}\mathbf{1}_p\right)\mathbf{\Omega}_h^{(r)^{-1}}\left(\boldsymbol{y}_j-\boldsymbol{\mu}_h^{(r)}\right)$$

and

$$\boldsymbol{V}_{4,hj}^{(r)}=\boldsymbol{\zeta}_h^{(r)}\mathbf{\Psi}_h\boldsymbol{\zeta}_h^{(r)}+\xi_{2h}^{(r)^2}\mathbf{1}_p\mathbf{1}_p^T.$$

It follows that $\boldsymbol{S}_{3,hj}^{(r)}$ and $S_{4,hj}^{(r)}$ are given by

$$\begin{aligned}
\boldsymbol{S}_{3,hj}^{(r)} \quad = \quad & \left(\mathbf{\Psi}_h^{(r)}-\mathbf{\Psi}_h^{(r)}\boldsymbol{\zeta}_h^{(r)^T}\mathbf{\Omega}_h^{(r)^{-1}}\boldsymbol{\zeta}_h^{(r)}\mathbf{\Psi}_h^{(r)}\right)\\
&+w_{hj}^{(r)}\mathbf{\Psi}_{hj}^{(r)}\boldsymbol{\zeta}_h^{(r)^T}\mathbf{\Omega}_h^{(r)^{-1}}(\boldsymbol{y}_j-\boldsymbol{\mu}_h^{(r)})(\boldsymbol{y}_j-\boldsymbol{\mu}_h^{(r)})^T\mathbf{\Omega}_h^{(r)^{-1}}\boldsymbol{\zeta}_h^{(r)}\mathbf{\Psi}_h^{(r)}\\
&+\mathbf{\Psi}_h^{(r)}\boldsymbol{\zeta}_h^{(r)^T}\mathbf{\Omega}_h^{(r)^{-1}}\boldsymbol{\delta}_h^{(r)}\boldsymbol{\delta}_h^{(r)^T}\mathbf{\Omega}_h^{(r)^{-1}}\boldsymbol{\zeta}_h^{(r)}\mathbf{\Psi}_h^{(r)}\boldsymbol{S}_{2,hj}^{(r)}\\
&-\mathbf{\Psi}_h^{(r)}\boldsymbol{\zeta}_h^{(r)}\mathbf{\Omega}_h^{(r)^{-1}}(\boldsymbol{y}_j-\boldsymbol{\mu}_h^{(r)})\boldsymbol{\delta}_h^{(r)T}\mathbf{\Omega}_h^{(r)^{-1}}\boldsymbol{\zeta}_h^{(r)}\mathbf{\Psi}_h^{(r)}\boldsymbol{S}_{1,hj}^{(r)}\\
&-\mathbf{\Psi}_h^{(r)}\boldsymbol{\zeta}_h^{(r)^T}\mathbf{\Omega}_h^{(r)^{-1}}\boldsymbol{\delta}_h^{(r)}(\boldsymbol{y}_j-\boldsymbol{\mu}_h^{(r)})^T\mathbf{\Omega}_h^{(r)^{-1}}\boldsymbol{\zeta}_h^{(r)}\mathbf{\Psi}_h^{(r)}\boldsymbol{S}_{1,hj}^{(r)}
\end{aligned} \tag{S52}$$

and

$$\begin{aligned}
\boldsymbol{S}_{4,hj}^{(r)} \quad = \quad & \left(\xi_{2h}^{(r)^2}-\xi_{2h}^{(r)^2}\mathbf{1}_p^T\mathbf{\Omega}_h^{(r)^{-1}}\mathbf{1}_p\right)\\
&+w_{hj}^{(r)}\xi_{2h}^{(r)^2}\mathbf{1}_p^{(r)^T}\mathbf{\Omega}_h^{(r)^{-1}}(\boldsymbol{y}_j-\boldsymbol{\mu}_h^{(r)})(\boldsymbol{y}_j-\boldsymbol{\mu}_h^{(r)})^T\mathbf{\Omega}_h^{(r)^{-1}}\mathbf{1}_p\\
&+\xi_{2h}^{(r)^2}\mathbf{1}_p^T\mathbf{\Omega}_h^{(r)^{-1}}\boldsymbol{\delta}_h^{(r)}\boldsymbol{\delta}_h^{(r)T}\mathbf{\Omega}_h^{(r)^{-1}}\mathbf{1}_p\boldsymbol{S}_{2,hj}^{(r)}\\
&-\xi_h^{(r)^2}\mathbf{1}_p^T\mathbf{\Omega}_h^{(r)^{-1}}\boldsymbol{\delta}_h^{(r)}(\boldsymbol{y}_j-\boldsymbol{\mu}_h^{(r)})^T\mathbf{\Omega}_h^{(r)^{-1}}\mathbf{1}_p\boldsymbol{S}_{1,hj}^{(r)}\\
&-\xi_h^{(r)^2}\mathbf{1}_p^T\mathbf{\Omega}_h^{(r)^{-1}}(\boldsymbol{y}_j-\boldsymbol{\mu}_h^{(r)})\boldsymbol{\delta}_h^{(r)^T}\mathbf{\Omega}_h^{(r)^{-1}}\mathbf{1}_p\boldsymbol{S}_{1,hj}^{(r)}.
\end{aligned} \tag{S53}$$

Further, we have that

$$\boldsymbol{S}_{5,hj}^{(r)} = \left[\boldsymbol{y}_j - \boldsymbol{\zeta}_h^{(r)}\mathbf{1}_p - \left(\boldsymbol{\zeta}_h^{(r)}\boldsymbol{\Psi}_h^{(r)}\boldsymbol{\zeta}_h^{(r)^T} + \xi_h^{(r)^2}\mathbf{1}_p\mathbf{1}_p^T\right)\boldsymbol{\Omega}_h^{(r)^{-1}}\left(\boldsymbol{y}_j - \boldsymbol{\mu}_h^{(r)}\right)\right]S_{1,hj}^{(r)}$$
$$+ \left(\boldsymbol{\zeta}_h^{(r)}\boldsymbol{\Psi}_h^{(r)}\boldsymbol{\zeta}_h^{(r)^T} + \xi_{2h}^{(r)^2}\mathbf{1}_p\mathbf{1}_p^T\right)\boldsymbol{\Omega}_h^{(r)^{-1}}\boldsymbol{\delta}_h^{(r)}S_{2,hj}^{(r)} \tag{S54}$$
$$\tag{S55}$$

and

$$\boldsymbol{S}_{6,hj}^{(r)} = \boldsymbol{V}_{4,hj}^{(r)} - \boldsymbol{V}_{4,hj}^{(r)}\boldsymbol{\Omega}_h^{(r)^{-1}}\boldsymbol{V}_{4,hj}^{(r)} + w_{hj}^{(r)}\boldsymbol{V}_{3,hj}^{(r)}\boldsymbol{V}_{3,hj}^{(r)^T}$$
$$- \boldsymbol{S}_{5,hj}^{(r)}\boldsymbol{\delta}_h^{(r)^T} - \boldsymbol{\delta}_h^{(r)}\boldsymbol{S}_{5,hj}^{(r)^T} + S_{2,hj}^{(r)}\boldsymbol{\delta}_h^{(r)}\boldsymbol{\delta}_h^{(r)^T}. \tag{S56}$$

To evaluate $\boldsymbol{S}_{7,hj}^{(r)}$, let $\boldsymbol{A}_{hj}$ be the diagonal matrix with $\boldsymbol{a}_{hj}$ as its diagonal elements. Then

$$E_{\boldsymbol{\Psi}^{(r)}}\left(\boldsymbol{A}_{hj}\boldsymbol{\Sigma}_h^{(r)^{-1}}\boldsymbol{A}_{hj} \mid \boldsymbol{y}_j, w_{hj}, u_{hj}\right) = E_{\boldsymbol{\Psi}^{(r)}}\left(\boldsymbol{a}_{hj}\boldsymbol{a}_{hj}^T \mid \boldsymbol{y}_j, w_{hj}, u_{hj}\right) \odot \boldsymbol{\Sigma}_h^{(r)^{-1}}. \tag{S57}$$

It is straightforward to show that

$$\begin{aligned}
\boldsymbol{S}_{7,hj}^{(r)} \;=\;& \Big\{\left(\boldsymbol{\Psi}_h^{(r)} - \boldsymbol{\Psi}_{ah}^{(r)}\boldsymbol{\zeta}_h^{(r)^T}\boldsymbol{\Omega}_h^{(r)^{-1}}\boldsymbol{\zeta}_h^{(r)}\boldsymbol{\Psi}_h^{(r)}\right)\\
+\;& w_{hj}^{(r)}\left[\mathbf{1}_p + \boldsymbol{\Psi}_h^{(r)}\boldsymbol{\zeta}_h^{(r)^T}\boldsymbol{\Omega}_h^{(r)^{-1}}\left(\boldsymbol{y}_j - \boldsymbol{\mu}_h^{(r)}\right)\right]\left[\mathbf{1}_p + \boldsymbol{\Psi}_h^{(r)}\boldsymbol{\zeta}_h^{(r)^T}\boldsymbol{\Omega}_h^{(r)^{-1}}\left(\boldsymbol{y}_j - \boldsymbol{\mu}_h^{(r)}\right)\right]^T\\
-\;& S_{1,hj}^{(r)}\left[\boldsymbol{\Psi}_h^{(r)}\boldsymbol{\zeta}_h^{(r)^T}\boldsymbol{\Omega}_h^{(r)}\boldsymbol{\delta}_h^{(r)}\right]\left[\mathbf{1}_p + \boldsymbol{\Psi}_h^{(r)}\boldsymbol{\zeta}_h^{(r)^T}\boldsymbol{\Omega}_h^{(r)^{-1}}\left(\boldsymbol{y}_j - \boldsymbol{\mu}_h^{(r)}\right)\right]^T\\
-\;& S_{1,hj}^{(r)}\left[\mathbf{1}_p + \boldsymbol{\Psi}_h^{(r)}\boldsymbol{\zeta}_h^{(r)^T}\boldsymbol{\Omega}_h^{(r)^{-1}}\left(\boldsymbol{y}_j - \boldsymbol{\mu}_h^{(r)}\right)\right]\left[\boldsymbol{\delta}_h^{(r)^T}\boldsymbol{\Omega}_h^{(r)^{-1}}\boldsymbol{\zeta}_h^{(r)}\boldsymbol{\Psi}_h^{(r)}\right]\\
+\;& S_{2,hj}^{(r)}\boldsymbol{\Psi}_h^{(r)}\boldsymbol{\zeta}_h^{(r)^T}\boldsymbol{\Omega}_h^{(r)^{-1}}\boldsymbol{\delta}_h^{(r)}\boldsymbol{\delta}_h^{(r)^T}\boldsymbol{\Omega}_h^{(r)^{-1}}\boldsymbol{\zeta}_h^{(r)}\boldsymbol{\Psi}_h^{(r)}\Big\} \odot \boldsymbol{\Sigma}_h^{(r)^{-1}}.
\end{aligned} \tag{S58}$$

To obtain $\boldsymbol{S}_{8,hj}^{(r)}$, observe that

$$\begin{aligned}
& E_{\boldsymbol{\Psi}^{(r)}}\left(\boldsymbol{A}_{hj}\boldsymbol{\Sigma}_h^{(r)^{-1}}\left(\boldsymbol{y}_j - \mathbf{1}_p b_{hj} - \boldsymbol{\delta}_h^{(r)}u_{hj}\right) \mid \boldsymbol{y}_j, w_{hj}, u_{hj}\right)\\
=\;& E_{\boldsymbol{\Psi}^{(r)}}\left(\boldsymbol{A}_{hj} \mid \boldsymbol{y}_j, w_{hj}, u_{hj}\right)\boldsymbol{\Sigma}_h^{(r)^{-1}}\left(\boldsymbol{y}_j - \boldsymbol{\delta}_h^{(r)}u_{hj}\right) - E_{\boldsymbol{\Psi}^{(r)}}\left(\boldsymbol{A}_{hj}b_{hj} \mid \boldsymbol{y}_j, w_{hj}, u_{hj}\right)\boldsymbol{\Sigma}_h^{(r)^{-1}}\mathbf{1}_p.
\end{aligned}$$

It follows that

$$\begin{aligned}
\boldsymbol{S}_{8,hj}^{(r)} \;=\;& \mathrm{DIAG}\left[\mathbf{1}_p + \boldsymbol{\Psi}_h^{(r)}\boldsymbol{\zeta}_h^{(r)^T}\boldsymbol{\Omega}_h^{(r)^{-1}}\left(\boldsymbol{y}_j - \boldsymbol{\mu}_h\right)\right]\boldsymbol{\Sigma}_h^{(r)^{-1}}\left(\boldsymbol{y}_j w_{hj}^{(r)} - S_{1,hj}^{(r)}\boldsymbol{\delta}_h^{(r)}\right)\\
& - \mathrm{DIAG}\left(\boldsymbol{\Psi}_h^{(r)}\boldsymbol{\zeta}_h^{(r)^T}\boldsymbol{\Omega}_h^{(r)}\boldsymbol{\delta}_h^{(r)}\right)\boldsymbol{\Sigma}_h^{(r)^{-1}}\left(S_{1,hj}^{(r)}\boldsymbol{y}_j - S_{2,hj}^{(r)}\boldsymbol{\delta}_h^{(r)}\right)\\
& + \mathrm{DIAG}\left(\boldsymbol{V}_{5,hj}^{(r)}\right)\boldsymbol{\Sigma}_h^{(r)^{-1}}\mathbf{1}_p,
\end{aligned} \tag{S59}$$

$$\begin{aligned}
\boldsymbol{V}_{5,hj}^{(r)} \;=\;& \xi_{2h}^{(r)^2}\boldsymbol{\Psi}_h^{(r)}\boldsymbol{\zeta}_h^{(r)}\boldsymbol{\Omega}_h^{(r)^{-1}}\mathbf{1}_p\\
& + w_{hj}^{(r)}\xi_{2h}^{(r)^2}\left[\mathbf{1}_p + \boldsymbol{\Psi}_h^{(r)}\boldsymbol{\zeta}_h^{(r)}\boldsymbol{\Omega}_h^{(r)^{-1}}\left(\boldsymbol{y}_j - \boldsymbol{\mu}_h^{(r)}\right)\right]\left(\boldsymbol{y}_j - \boldsymbol{\mu}_h^{(r)}\right)^T\boldsymbol{\Omega}_h^{(r)^{-1}}\mathbf{1}_p\\
& - \boldsymbol{S}_{1,hj}^{(r)}\xi_{2h}^{(r)^2}\left[\mathbf{1}_p + \boldsymbol{\Psi}_h^{(r)}\boldsymbol{\zeta}_h^{(r)^T}\boldsymbol{\Omega}_h^{(r)^{-1}}\left(\boldsymbol{y}_j - \boldsymbol{\mu}_h^{(r)}\right)\right]\boldsymbol{\delta}_h^{(r)}\boldsymbol{\Omega}_h^{(r)^{-1}}\mathbf{1}_p\\
& - \boldsymbol{S}_{1,hj}^{(r)}\xi_{2h}^{(r)^2}\mathbf{1}_p^T\boldsymbol{\Omega}_h^{(r)^{-1}}\boldsymbol{\delta}_h^{(r)T}\left[\left(\boldsymbol{y}_j - \boldsymbol{\mu}_h^{(r)}\right)^T\boldsymbol{\Omega}_h^{(r)^{-1}}\boldsymbol{\zeta}_h^{(r)}\boldsymbol{\Psi}_h^{(r)} + \mathbf{1}_p^T\right]\\
& + \boldsymbol{S}_{2,hj}^{(r)}\xi_{2h}^{(r)^2}\mathbf{1}_p^T\boldsymbol{\Omega}_h^{(r)^{-1}}\boldsymbol{\delta}_h^{(r)}\boldsymbol{\delta}_h^{(r)T}\boldsymbol{\Omega}_h^{(r)^{-1}}\mathbf{1}_p.
\end{aligned} \tag{S60}$$

2.2 M-step

The estimates of the parameters are updated on the M-step by maximizing the Q-function over the parameter space. It follows that

$$\pi_h^{(r+1)} = \frac{1}{n} \sum_{j=1^n} z_{hj}^{(r)}, \tag{S61}$$

$$\boldsymbol{\Psi}_h^{(r+1)} = \frac{\sum_{j=1}^n z_{hj}^{(r)} \boldsymbol{S}_{3,hj}^{(r)}}{\sum_{j=1}^n z_{hj}^{(r)}}, \tag{S62}$$

$$\xi_{2h}^{(r+1)^2} = \frac{\sum_{j=1}^n z_{hj}^{(r)} S_{4,hj}^{(r)}}{\sum_{j=1}^n z_{hj}^{(r)}}, \tag{S63}$$

$$\boldsymbol{\delta}_h^{(r+1)} = \frac{\sum_{j=1}^n z_{hj}^{(r)} \boldsymbol{S}_{5,hj}^{(r)}}{\sum_{j=1}^n z_{hj}^{(r)} S_{2,hj}^{(r)}}, \tag{S64}$$

$$\boldsymbol{\Sigma}_h^{(r+1)} = \frac{\sum_{j=1}^n z_{hj}^{(r)} \boldsymbol{S}_{6,hj}^{(r)}}{\sum_{j=1}^n z_{hj}^{(r)}}, \tag{S65}$$

and

$$\boldsymbol{\mu}_h^{(r+1)} = \left(\sum_{j=1}^n z_{hj}^{(r)} \boldsymbol{S}_{7,hj}^{(r)} \right)^{-1} \left(\sum_{j=1}^n z_{hj}^{(r)} \boldsymbol{S}_{8,hj}^{(r)} \right). \tag{S66}$$

The update of the degrees of freedom $\nu_h^{(r+1)}$ is given implicitly as a solution of the equation,

$$\frac{\sum_{j=1}^n z_{hj}^{(r)} \left[\psi\left(\frac{\nu_h^{(r)}+p}{2} \right) - \log\left(\frac{\nu_h^{(r)}+d_h^{(r)}(\boldsymbol{y}_j)}{2} \right) - \frac{\nu_h^{(r)}+p}{\nu_h^{(r)}+d_h^{(r)}(\boldsymbol{y}_j)} \right]}{\sum_{j=1}^n z_{hj}^{(r)}} + \log\left(\frac{\nu_h}{2}\right) - \psi\left(\frac{\nu_h}{2}\right) + 1 = 0. \tag{S67}$$

where $\psi(\cdot)$ denotes the Digamma function.
