# Supplementary material for: Joint Modeling and Registration of Cell Populations in Cohorts of High-Dimensional Flow Cytometric Data
Source: PLoS One. 2014 Jul 1;9(7):e100334. doi: 10.1371/journal.pone.0100334 (PMC4077578; doi:10.1371/journal.pone.0100334)
Supplement: Text S1 — Data and experiments. (PDF) [file pone.0100334.s011.pdf]

Text S1 Data and experiments

Flow cytometry is one of the most popular bio-technological platforms that are used in the clinical and the research labs worldwide. It provides per cell data on the expression levels of markers on the surface and within cells, which helps in characterization of cell populations, and to determine their sizes, types and functions. This has made flow cytometry highly useful in the understanding of infectious diseases, cancers, and a number of increasingly advanced bio-medical applications. Below we describe two typical examples of rich flow cytometric data that are generated across multiple time-points or multiplexed panels of markers. A figure graphically explaining the design of such data generation given in Irish et al. (Fig. 1A in [1]).

1.1 TCR stimulation time course data

Flow-cytometry based assessment of T cell phosphorylation patterns in healthy human subjects was performed previously [2]. While the experiments were originally conducted by the Maier et al. study [2], it is for the first time that the time course data for all six classes are being analyzed using automaton, and as high-dimensional data, by the present study. Briefly, whole blood samples from healthy Caucasian individuals were obtained with informed consent and according to the Institute Ethics Review Board protocols. Here we use the samples from three individuals that were studied over a time course of six time points: 0 min (pre-stimulation) and 1, 3, 5, 15, and 30 min (post-stimulation). For four-color cell surface and intercellular staining, 250,000 cells in each sample were stained using labeled antibodies CD4, CD45RA, SLP76(pY128), and ZAP70(pY292) before T cell receptor stimulation with an anti-CD3 antibody (baseline measurement; 0 min). All samples were acquired on a FACS Calibur (BD Biosciences) machine using CellQuest software within 12 hours after staining. Live cell gating was performed by Maier et al. [2] using the flowJo software. As part of data preprocessing for the present study, logicle transformation (using the flowCore package [3]) was applied to every sample; see Maier et al. [2] for further details.

1.2 BCR signaling data from follicular lymphoma cohorts

Follicular lymphoma (FL) tumor samples were acquired before any therapy from newly diagnosed 28 patients. All specimens were obtained with informed consent in accordance with the Declaration of Helsinki and this study was approved by Stanford University's Administrative Panels on Human Subjects in Medical Research. Tumor signaling heterogeneity was analyzed using phospho-specific flow cytometry as described in detail in the Supplemental Methods of Irish et al. [1]. Basal levels of signaling in unstimulated cells (0 minutes) were used to examine constitutive or tonic signaling. The BCR signaling response of 15 phospho-proteins was calculated as fold induction of signaling over basal at 4 minutes following stimulation by F(ab')2 against the BCR heavy chain. Lymphoma Negative Prognostic (LNP) cells, a subpopulation of lymphoma B cells found at diagnosis in some tumors, were previously shown to be continuously associated with poor overall survival [1]. The samples analyzed here constituted the Testing Set of 28 FL samples from this prior study. Three post-therapy samples from Irish et al. [1] were included to assess the effect of any inter-sample variation on JCM modeling. The 28 samples are analyzed in high dimension for the first time in the current study. LNP cells were previously quantified in these samples based on an impaired BCR signaling response in a subset of lymphoma B cells with distinct CD20 and BCL2 expression (Supplemental Methods of Irish et al. [1]). Each stimulation condition for a FL patient sample was split for staining with one of eight staining panels which each measured a pair of phospho proteins (e.g. p-PLCg and p-STAT5) plus the lineage markers CD3, CD5, CD20, and BCL2 and cell light scatter properties. Using eight panels allowed us to measure 16 phospho-proteins, but meant that on a given cell we only measured two phospho-proteins simultaneously. To overcome this, CD20 and BCL2 expression were used to correlate subpopulations of FL cells between staining panels [1].

References

1. Irish, J. M. et al. B-cell signaling networks reveal a negative prognostic human lymphoma cell subset that emerges during tumor progression. Proc Natl Acad Sci U S A 107, 12747-12754, (2010).

2. Maier, L. M., Anderson, D. E., De Jager, P. L., Wicker, L. S. & Hafler, D. A. Allelic variant in CTLA4 alters T cell phosphorylation patterns. Proc Natl Acad Sci U S A 104, 18607-18612, (2007).

3. Hahne, F. et al. flowCore: a Bioconductor package for high throughput flow cytometry. BMC Bioinformatics 10, 106, (2009).
