# Supplementary material for: Joint Modeling and Registration of Cell Populations in Cohorts of High-Dimensional Flow Cytometric Data
Source: PLoS One. 2014 Jul 1;9(7):e100334. doi: 10.1371/journal.pone.0100334 (PMC4077578; doi:10.1371/journal.pone.0100334)
Supplement: Text S2 — The JCM workflow. (PDF) [file pone.0100334.s012.pdf]

Text S2 The JCM workflow

Step 1: JCM input: The input to JCM is a batch of m flow cytometric samples in the form of either a zipped folder of m .txt or .csv files. We assume that the flow data have been acquired, quality-controlled and preprocessed (such as live cell gating) properly. Commercial software and freely available BioConductor packages (e.g. flowCore [1]) are highly useful for such purposes. If q (>1) multiplexed panels of markers were used, then q such zipped folders or flowSet objects matrices must be provided, such that every sample is represented by q panels. The user can also specify which mixture model (MT or MST) to fit, and an optional range for the expected number of populations (g) in the batch template.

For each sample k, we extract from its .fcs file or R flowFrame an n_k by p expression matrix, corresponding to fluorescence intensity values of p markers or antibodies for n_k cells. (Typically, p varies between 4 and 8 but could be as high as 17 in fluorescence cytometry and 35-40 in mass cytometry, but JCM is not limited by any particular value of p; n_k could range from hundreds to hundreds of thousands per sample; q is currently a moderate constant such as 10 or less whereas m could be in hundreds.)

Step 2: Multi-level modeling: Details of the models are given in the Methods section of the main paper. We used the EM algorithm of Dempster et al. [2] to fit the g-component t- and skew t-mixture model with component distributions defined by (2) and (5), respectively. The fitting of mixtures of t-distributions [3] is explained in some detail in McLachlan and Peel [4]. It is computationally convenient to use the characterization (4) to define the EM framework for the fitting of a mixture of these skew t-densities. However, the E-step involves the calculation of a number of conditional expectations that cannot be be expressed in closed form. Previously, Pyne et al. [5] circumvented this problem by replacing the term $\boldsymbol{\Delta}_h|\boldsymbol{U}|$ in (4) by the term $|U|\boldsymbol{\delta}_h$, where U is a standard univariate normal random variable. With this simplification, the skew t-density (5) reduces to

$$2t_p(\boldsymbol{y};\boldsymbol{\mu}_h,\boldsymbol{\Omega}_h,\nu_h)T_1(y^*;0,1,\nu_h+p), \tag{S1}$$

where $\boldsymbol{y}^* = [(\nu_h+p)/\{\nu_h+d_h(\boldsymbol{y})\}\{1-\boldsymbol{\delta}_h^T\boldsymbol{\Omega}_h^{-1}\boldsymbol{\delta}_h\}^{-1}]^{1/2}\boldsymbol{\delta}_h^T\boldsymbol{\Omega}_h^{-1}(\boldsymbol{y}-\boldsymbol{\mu}_h)$. For the simplified form (1) of the skew t-density, the calculations on the E-step can be expressed in closed form. For the data sets analysed by Pyne et al. [5] this simplification appeared to make little difference in the fit provided by the mixture of skew t-distributions so modified. Hence for computational convenience we shall continue to work here with this simplified form.

In the framework of the EM algorithm for the fitting of this proposed multilevel mixture model, the complete-data are taken to be the observed samples together with the unobservable data corresponding to the variables w, U, and $\boldsymbol{U}_0$ in the characterization (4) for the skew t-distribution, and the unknown component labels for the individual observations (cells). As observations from the same sample share common random effects terms, they will not be independently distributed. However, they will be independent conditional on the random effects, and so the complete-data log likelihood can be formed in a straightforward way for the application of the EM algorithm. Some of the conditional expectations in the E-step cannot be calculated in closed form bearing in mind that there are multiplicative random effects terms in (6) for scaling variation. We therefore proceeded by taking the multiplicative random effects to be cell-specific, i.e. using a_{hijk} instead of a_{hik} in (6). If the additive random effects terms were taken to be cell-specific, then they would be no longer identifiable from the error term and, so to avoid this, while they are taken to be cell-specific they are taken to be the same for each marker $i (i = 1,\ldots,m)$; that is, b_{hik} in (6) is replaced by b_{hjk}. This means that the random-effects terms are distributed as

$$a_{hijk} \sim N(1,\xi_{1hi}^2) \quad (i=1,\ldots,p) \quad \text{and} \quad b_{hjk} \sim N(0,\xi_{2h}^2) \tag{S2}$$

for a given h, j, and k $(h = 1, \ldots, g;\ j = 1, \ldots, n_k;\ k = 1, \ldots, m)$.

The E- and M-steps, as described in Appendix A, are alternately repeated until the relative change in log likelihood is smaller than 10^{-6}, or the number of iterations reaches 1000, whichever happens earlier. If a range for the number of populations (g) in the template is not specified, JCM precomputes one using the average silhouette width (implemented by pamk from the R package fpc). The selection of the optimal number of components in the mixture model is made by default on the basis of the Bayesian Information Criterion (BIC), although other criteria can be used.

Step 3: Output files: A list of all the input parameters ("input_parameters.txt") used for running JCM is included in the output. A tab-separated file ("batch_features.txt") contains the JCM features for every modeled sample in the batch such that each row represents a fitted parameter of the models and each column a sample. The population indices are matched across samples. The same information is also provided in GenePattern compatible format ("batch_features.gct"). A second tab-separated file ("template_features.txt") contains the features of the batch template. The template features are also provided as an R object ("template_model.ret") which can be read using the dget() function. A default list of feature-sets ("featuresets_default.gmt") is generated by pooling JCM features in all panels and grouping them by types, such as population means, proportions, etc. An overlay plot of all templates across different classes ("template_overlay.pdf") and one for each class showing within-class variation ("class_overlay.pdf") are produced. Fluorescence intensity heatplots are produced for side-by-side spatial or temporal comparison of individual populations that are matched across all samples. The option of pairplots to illustrate the mixture model based clustering for each sample is also available ("sample_pairplot.pdf"). Finally, JCM outputs a zipped folder (cluster_labels.txt") containing the cluster-membership labels for each point in every sample of the batch.

Step 4: Downstream analysis: JCMs output formats allow easy application of visualization and other analytical routines in R, GenePattern, and other platforms. In the present analysis, for the two-class BCR signaling data, we used the Gene Set Enrichment Analysis module of GenePattern to identify the enriched meta-features (or feature-sets) across multiplexed staining panels. Although we used the featuresets_default.gmt file, in general, the user can customize feature-sets by pooling specific JCM features from selected panels and grouping them probably with the help of such feature-attributes as type, cluster # or panel #, and save the result as a .gmt file. The customization can be done easily with any text editor. (Note, both the .gct and .gmt formats are described under GenePattern file formats.) These files can then be used as input to the GSEA to identify enriched cross-panel feature-sets. Using R, BioConductor or GenePattern, one can visualize the features of interest (say, population means), or indeed all features, as heatmaps, and also select the most distinctive ones for further investigation.

References

1. Hahne, F. et al. flowCore: a Bioconductor package for high throughput flow cytometry. BMC Bioinformatics 10, 106, (2009).

2. Dempster, A.P., Laird, N.M. & Rubin, D. Maximum likelihood from incomplete data via the EM algorithm. J. Royal Statist. Soc., B, 39, 1-38 (1977).

3. McLachlan, G.J. & Peel, D. Robust cluster analysis via mixtures of multivariate t-distribtuions. in Lecture Notes in Computer Science 1451, 658-666 (Springer-Verlag, 1998).

4. McLachlan, G.J. & Peel, D. Finite Mixture models. (Wiley, 2000).

5. Aghaeepour, N., Nikolic, R., Hoos, H. H. & Brinkman, R. R. Rapid cell population identification in flow cytometry data. Cytometry A 79, 6-13, (2011).
